# Probabilistic logic analysis of the highly heterogeneous spatiotemporal HFRS incidence distribution in Heilongjiang province (China) during 2005-2013

**DOI:** 10.1371/journal.pntd.0007091

**Published:** 2019-01-31

**Authors:** Junyu He, George Christakos, Jiaping Wu, Piotr Jankowski, Andreas Langousis, Yong Wang, Wenwu Yin, Wenyi Zhang

**Affiliations:** 1 Ocean College, Zhejiang University, Zhoushan, China; 2 Department of Geography, San Diego State University, San Diego, California, United States of America; 3 Department of Civil Engineering, University of Patras, Patras, Greece; 4 Chinese PLA Center for Disease Control and Prevention, Beijing, China; 5 Division of Infectious Diseases, Key Laboratory of Surveillance and Early-warning on Infectious Disease, Chinese Center for Disease Control and Prevention, Beijing, China; University of California Berkeley, UNITED STATES

## Abstract

**Background:**

Hemorrhagic fever with renal syndrome (HFRS) is a zoonosis caused by hantavirus (belongs to Hantaviridae family). A large amount of HFRS cases occur in China, especially in the Heilongjiang Province, raising great concerns regarding public health. The distribution of these cases across space-time often exhibits highly heterogeneous characteristics. Hence, it is widely recognized that the improved mapping of heterogeneous HFRS distributions and the quantitative assessment of the space-time disease transition patterns can advance considerably the detection, prevention and control of epidemic outbreaks.

**Methods:**

A synthesis of space-time mapping and probabilistic logic is proposed to study the distribution of monthly HFRS population-standardized incidences in Heilongjiang province during the period 2005–2013. We introduce a class-dependent Bayesian maximum entropy (cd-BME) mapping method dividing the original dataset into discrete incidence classes that overcome data heterogeneity and skewness effects and can produce space-time HFRS incidence estimates together with their estimation accuracy. A ten-fold cross validation analysis is conducted to evaluate the performance of the proposed cd-BME implementation compared to the standard class-independent BME implementation. Incidence maps generated by cd-BME are used to study the spatiotemporal HFRS spread patterns. Further, the spatiotemporal dependence of HFRS incidences are measured in terms of probability logic indicators that link class-dependent HFRS incidences at different space-time points. These indicators convey useful complementary information regarding intraclass and interclass relationships, such as the change in HFRS transition probabilities between different incidence classes with increasing geographical distance and time separation.

**Results:**

Each HFRS class exhibited a distinct space-time variation structure in terms of its varying covariance parameters (shape, sill and correlation ranges). Given the heterogeneous features of the HFRS dataset, the cd-BME implementation demonstrated an improved ability to capture these features compared to the standard implementation (e.g., mean absolute error: 0.19 *vs*. 0.43 cases/10^5^ capita) demonstrating a point outbreak character at high incidence levels and a non-point spread character at low levels. Intraclass HFRS variations were found to be considerably different than interclass HFRS variations. Certain incidence classes occurred frequently near one class but were rarely found adjacent to other classes. Different classes may share common boundaries or they may be surrounded completely by another class. The HFRS class 0–68.5% was the most dominant in the Heilongjiang province (covering more than 2/3 of the total area). The probabilities that certain incidence classes occur next to other classes were used to estimate the transitions between HFRS classes. Moreover, such probabilities described the dependency pattern of the space-time arrangement of HFRS patches occupied by the incidence classes. The HFRS transition probabilities also suggested the presence of both positive and negative relations among the main classes. The HFRS indicator plots offer complementary visualizations of the varying probabilities of transition between incidence classes, and so they describe the dependency pattern of the space-time arrangement of the HFRS patches occupied by the different classes.

**Conclusions:**

The cd-BME method combined with probabilistic logic indicators offer an accurate and informative quantitative representation of the heterogeneous HFRS incidences in the space-time domain, and the results thus obtained can be interpreted readily. The same methodological combination could also be used in the spatiotemporal modeling and prediction of other epidemics under similar circumstances.

## Author summary

Heilongjiang Province reported the largest number of HFRS cases in China. Previous studies focused on identifying the severe HFRS outbreak regions, exploring the relative impact of environmental factors, forecasting HFRS cases etc. However, the study of the spatiotemporal spread dynamics and patterns of HFRS is still lacking, which is the focus of the present study. This study proposed a novel mapping technique (i.e., class-dependent Bayesian Maximum Entropy, cd-BME) for studying the distribution of HFRS, overcoming the highly heterogeneous features of HFRS data; and, probabilistic logic notions (stochastic indicators) were employed to study the spatiotemporal dependency of HFRS incidence and draw conclusions regarding the HFRS spread under conditions of uncertainty. By dividing the original HFRS data into four classes in terms of percentiles, the cd-BME exhibited better performance in mapping HFRS distribution than the standard (class-independent) BME technique and the mainstream inverse distance technique. Regarding the maps of HFRS distribution, the point outbreak character dominated the HFRS spread at high incidence levels, whereas the lowest incidence level covered more than 2/3 of Heilongjiang Province. Certain HFRS incidence generally occurred between intraclass or neighbor classes. The probabilities of HFRS transition between incidence classes with various spatial distances and temporal instants can be found in the HFRS indicator plots. The above comprehensive information can allow a better understanding of the spatiotemporal HFRS spread mechanisms and further improve HFRS decision-making, management and control.

## Introduction

The first cases of hemorrhagic fever with renal syndrome (HFRS) were reported in northeastern China in the early 1930s [1]. During the past seven decades, this rodent-borne zoonosis, caused by Hantavirus, has been spreading southwards to other parts of China. Currently, 31 provinces, autonomous regions, and metropolitan areas of mainland China, are exposed to significant health risks due to this infectious disease. In particular, the reported HFRS cases correspond to approximately 90% of all global number of cases [2, 3]. Specifically, the *Hantaan* virus (HTNV) and the *Seoul* virus (SEOV), hosted by *Apodemus agrarius* and *Rattus norvegicus*, respectively, are the two predominant sources of HFRS in China (see, e.g., [1, 4, 5]). The hantavirus is transmitted from rodents to humans through inhalation of aerosolized excreta (such as urine and saliva) or direct contact [6]. Infected human specimens suffer from fever, headache, abdominal pain, insufficient renal function, and hemorrhagic episodes [7, 8]. For the period 2004–2008, most HFRS cases reported in China concerned young and middle-age farmers [9] with case fatality rate (CFR) 1.17%, and females experiencing higher CFRs than males in the age groups of 20–39 and >50 years [10]. Also HFRS cases in males were more than three times higher than those reported in females. Evidently, HFRS poses a serious threat to public health in China.

Previous HFRS studies have focused on different aspects of the epidemic, such as the following cases:

1. Identification of regions with severe HFRS outbreaks. Zhang et al. [2], e.g., used spatial autocorrelation, local indicators of spatial association, and Kulldorff’s space-time scan statistic, to identify distinct cluster areas of outbreak episodes in northeastern, central and eastern China. Similarly, Wu et al. [11] applied cluster analysis, temporal cluster analysis, and space-time cluster analysis, to identify various epidemic clusters in Liaoning Province of China (including a primary cluster in the western part of the province and two secondary clusters in its eastern part).
2. Quantitative analysis of the impact of physiographic characteristics and environmental variables on HFRS population-standardized incidences. Yan et al. [12], e.g., employed a multivariate logistic regression model to study the relationship between HFRS incidence and various landscape and environmental elements. The study concluded that elevation, Normalized Difference Vegetation Index (NDVI), precipitation, annual cumulative air temperature, semihydromorhic soils, timber forests and orchards, were closely related to HFRS incidence. In a more recent study, Li et al. [13] applied a geographically weighted regression model to Chinese data during the period 2005–2009, and concluded that temperature, precipitation, humidity, NDVI, land use and elevation significantly impacted the spatiotemporal heterogeneity of HFRS incidences. Also, Tian et al. [14] and He et al. [15, 16] used wavelet analysis to study how the dynamics of HFRS are linked to rainfall, temperature, rodents’ density and the multivariate El Niño-Southern Oscillation (ENSO) index.
3. HFRS incidence forecasting. Liu et al. [17] and Li et al. [18] successfully applied autoregressive integrated moving average (ARIMA) and seasonal ARIMA models to produce forecasts of HFRS in China and Heilongjiang Province, respectively. Moreover, seasonal ARIMA models with exogenous variables (SARIMAX) were also developed for forecasting HFRS in four counties with severe HFRS outbreaks [19].
4. Clinical manifestation case studies. For example, Zhang et al. [20] compared various clinical indices involving 152 patients in Heilongjiang Province, and showed that SEOV infections are milder and less typical than those caused by HTNV.

While, as described above, a significant level of understanding has been reached regarding the specific characteristics of HFRS and its linkage to physiographic variables, its dynamics and associated spatiotemporal spread patterns remain mostly unexplored. The present work aims at bridging this gap, by using the spatiotemporal Bayesian Maximum Entropy theory (BME) to develop a fully probabilistic approach that allows a rigorous quantitative representation of HFRS population-standardized incidences in the space-time domain (BME belongs to the field of modern spatiotemporal geostatistics, [21, 22]). At this point we notice that BME has been successfully applied in the study of several infectious diseases, such as syphilis, hand food and mouth disease, influenza, dengue fever, and Black death [23–28]. Our study area is the Heilongjiang Province, which experiences the highest HFRS population-standardized incidences in China [2, 12, 18], caused by both HTNV and SEOV [20]. In the Heilongjiang case study, the main advantage of the proposed BME-based approach is that it can account for certain crucial aspects of space-time HFRS spread, as follows:

i) Spatiotemporal evolution: The proposed approach not only accounts for the fact that, as is the case with all infectious diseases, the spatiotemporal HFRS evolution can be influenced by several environmental factors, but also for the fact that, as a natural phenomenon, the HFRS spread follows a propagation law with the number of human infections being closely related to human-rodents interactions. In this twofold phenomenological context, when a core area is formed during the disease outbreak, and as time advances, the disease spreads radially from the core center in all directions. The accurate characterization of the spatiotemporal HFRS spread pattern is of essence, and the thus acquired knowledge can be integrated with other information sources (about the disease and its environment) to improve public health management through prevention and control.
ii) Incidence heterogeneity: A crucial observation to be accounted by the proposed approach is that the HFRS infection level varies in a heterogeneous manner across space-time, which means that the adequate distinction between incidence classes can play a significant role in the accurate mapping and risk assessment of regional disease spread during the time period of interest. Furthermore, since infectious disease outbreaks can occur in very short time periods and infect a large number of individuals, and given the highly variable character of disease attributes, it is difficult (both theoretically and practically) and often unrealistic to model simultaneously both the extreme values and the regular values of population-standardized incidences. In other words, a model that considers the distribution of all incidence levels as a single (super) class can neither adequately represent nor fully explain the spread pattern of a specific incidence class, because of smoothing effects and the existence of extreme (high/low) incidence levels in the same setting.
iii) Class transmission and propagation: The probabilistic assessment of the HFRS transmission and propagation patterns among various incidence classes is currently lacking (e.g., the probability of HFRS transmitting from one incidence class to another class at various spatial distances and time separations apart). Yet such an assessment could provide valuable scientific support for HFRS monitoring and control purposes. Accordingly, the adequate representation of the spatiotemporal distributions and operations (transmissions, propagations) between of distinct incidence classes is an important task of any quantitative HFRS study.

In view of the above considerations *i*-*iii*, the three main objectives of the present work are as follows:

1. To overcome data-related methodological and practical difficulties (such as highly heterogeneous data distributions across space-time) by using a cd-BME method that divides the infectious disease dataset into discrete classes (the incidence classes were selected based on variability reduction and mapping coverage criteria).
2. To generate HFRS distribution maps that account for the real fact that the intraclass and interclass incidence data is subject to uncertainty, and to show that the cd-BME method can produce more accurate incidence estimates at locations and times where no HFRS records are available than standard (class-independent) mapping techniques.
3. To introduce practical and easily interpretable indicators of spatiotemporal dependency based on probability logic [29]. These indicators exhibit different yet complementary kinds of HFRS dependence (e.g., each indicator captures a different feature of HFRS transition from one class to another so that the combined study of the indicators can result to an improved assessment of HFRS distribution).

## Materials and methods

### Ethics statement

The present study was approved by (*a*) the Chinese Center for Disease Control and Prevention, and (*b*) the Institute of Disease Control and Prevention. All HFRS data were anonymously analyzed.

### The HFRS data set and its categorization

**Fig 1:**
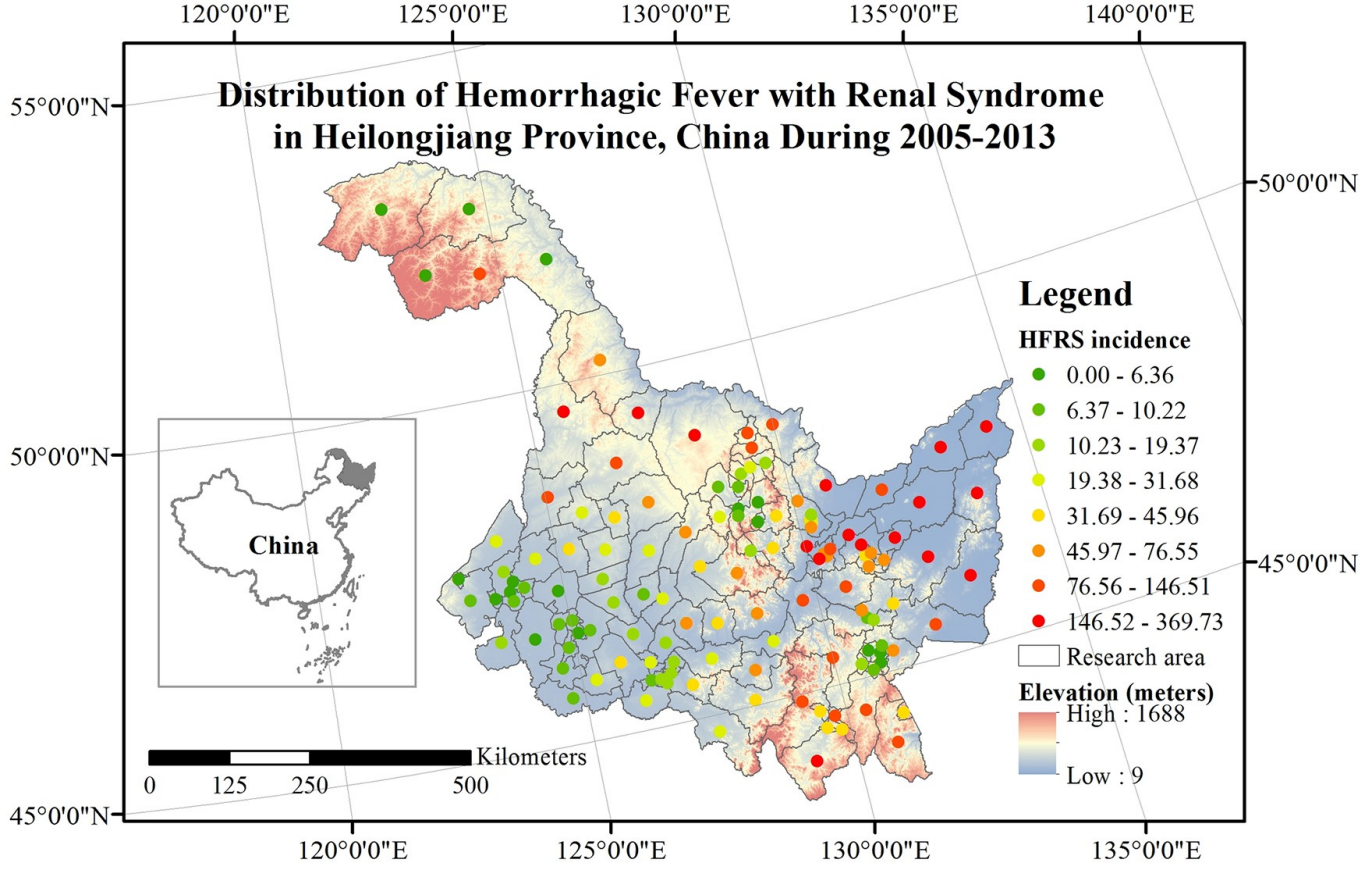
Population-standardized HFRS incidences for the period 2005–2013 (i.e. total incidences/10^5^ capita), in the 130 counties of the Heilongjiang Province in China (dots correspond to country centroids).

During the period January 2005–December 2013, China’s Information System for Disease Control and Prevention (CISDCP) recorded HFRS cases at 130 counties of the Heilongjiang province, with an approximate area of 473 thousand *km*^2^, and population of 38.35 million. During this period, the monthly rainfall, temperature and relative humidity ranged from 0.23 to 221.4 *mm*, -23.12 to 23.12 °*C* and 38.77 to 83.74%, respectively. The Heilongjiang Province has 38.98, 26.29 and 16.92% croplands, mixed forests and cropland/natural vegetation mosaic, respectively [15]. In addition, the GDP of Heilongjiang Province increased from 551.4 to 1445.5 billion *yuan*. The monthly HFRS case data were population-standardized using the corresponding demographic data obtained from the National Bureau of Statistics of China. Fig 1 shows the spatial distribution of HFRS population-standardized incidences during the entire study period (i.e., January 2005–December 2013) in the 130 counties of the Heilongjiang Province. The maps in the current study were all made by using the ArcGIS 10.2 software.

The space-time distribution of population-standardized HFRS incidences in Heilongjiang province during the period 2005–2013 are quantitatively represented using a spatiotemporal random field model[22], *X*(***p***), where ***p*** = (***s***, *t*) denotes the spatial coordinate ***s*** = (*s*_1_, *s*_2_) and time instant *t* (S1 Text). Also, ${Ι}_{m}=[\zeta_{l}^{m},\zeta_{u}^{m}]$ and ${Ι}_{n}=[\zeta_{l}^{n},\zeta_{u}^{n}]$ denote the selected HFRS incidence interval classes at the space-time points ***p*** = (***s***, *t*) and ***p*′** = (***s*′**, *t***′**), respectively, where the subscripts *m*, *n* = 1, 2, 3, 4 are the class identification numbers, and the subscripts *l* and *u* denote the lower and upper limit, respectively, of each class interval. Then, these class-based (categorical) HFRS incidences can be denoted as *X*(***p***) ∈ *I*_*m*_, which means that the HFRS incidence at point ***p*** belongs to the interval class *I*_*m*_, or *X*(***p***′) ∈ *I*_*n*_, which means that the incidence at point ***p***′ belongs to the class *I*_*n*_ (S2 Text). Given the high spatiotemporal variability of HFRS incidence values during the period 2005–2013 (the skewness is 6.265 and the kurtosis is 64.007), a twofold methodological choice was made seeking both variability reduction, space-time points coverage and mapping accuracy (S3 Text). In particular:

1. The data was log-transformed by means of *Y*(***p***) = log_10_(*X*(***p***) + 1). The additional advantage of this equation being that it can also account for zero values (such zero values are noteworthy since they may offer important clues to physical characteristics linked to the absence of disease or individual immunity).
2. The HFRS data set was categorized into four classes in terms of percentiles, as shown in Table 1, i.e., considering that 58% of the original data consist of 0 values, we divided the remaining 42% of the data into four classes by percentile (so that each of them has 10.5% of the data), and, subsequently all 0s (58%) were added to the first class, i.e., 0–68.5%, 68.5–79%, 79–89.5% and 89.5–100%. The selection of four classes was based on the satisfaction of the following quantitative criteria: (*i*) the variability criterion (i.e., after dividing the original data into a certain number of categories, the incidence variability should be reduced); (*ii*) the coverage criterion (i.e., the data coverage across the entire study area should decrease with increasing number of categories, and it is suggested to have data that covers more than 60% of the study area); and (*iii*) the mapping criterion (i.e., based on empirical considerations, to assure mapping accuracy a rigorous technique requires that a certain number of space-time data points around the estimation point should exist in its category).

**Table 1:** The four HFRS incidence classes and their descriptive statistics.

*Class* (*I*_*m*_) *no*.	*Percentile*	*Original dataset X*(*p*)		*Log-Transformed data set Y*(*p*)	
		*Lower limit* ($\zeta_{l}^{m}$)	*Upper limit* ($\zeta_{u}^{m}$)	*Lower limit*	*Upper limit*
1 (*I*_1_)	0–68.5	0 ($\zeta_{l}^{1}$)	0.3576 ($\zeta_{u}^{1}$)	0	0.1328
2 (*I*_2_)	68.5–79	0.3577 ($\zeta_{l}^{2}$)	0.6830 ($\zeta_{u}^{2}$)	0.1329	0.2261
3 (*I*_3_)	79–89.5	0.6831 ($\zeta_{l}^{3}$)	1.5277 ($\zeta_{u}^{3}$)	0.2262	0.4027
4 (*I*_4_)	89.5–100	1.5278 ($\zeta_{l}^{4}$)	26.4884 ($\zeta_{u}^{4}$)	0.4032	1.4439

*Note*: Units of min and max of original data are cases/10^5^ capita.

More specifically, as regards criterion *i*, due to the high HFRS data variability exhibiting a heavy-tailed distribution, it was found that by classifying the data in terms of incidence percentiles, the intraclass data variability was reduced significantly. In response to criterion *ii*, the particular incidence classes were selected because they allowed a sufficient number of space-time points in each class for further processing (HFRS mapping and indicator analysis). Within these interval classes *I*_1_, *I*_2_, *I*_3_ and *I*_4_ (Table 1) there existed, respectively, 9617 space-time points with 130 overlapping locations, 1470 points with 90 locations, 1478 points with 101 locations, and 1475 points with 96 locations. Hence, in the present study there were at least 90 locations in each incidence class (which can be regarded as a spatial data coverage criterion for improved mapping purposes). As regards criterion *iii*, based on empirical considerations a rigorous mapping technique requires that a certain number of space-time data points around the estimation point should exist in its category for mapping accuracy purposes.

In relation to the above, two notions can be used to describe quantitatively the HFRS pattern across space-time: the global size of each incidence class, and the spatiotemporal arrangement of the different incidence classes relative to each other. Individual HFRS incidence classes may visually appear to occupy mutually exclusive “patches” of various sizes within the space-time domain of interest (e.g., any pair of classes *I*_*m*_ and *I*_*n*_ may have or may have not common boundaries). These patches may be spread uniformly throughout the domain of interest, or they may appear to be elongated along a particular direction, in which case the HFRS pattern will be characterized as anisotropic. The distribution pattern of incidence classes is determined by their spatiotemporal dependence, which makes the latter a key notion of a quantitative HFRS study.

### Space-time mapping of HFRS incidence distribution using the variant BME method

**Fig 2:**
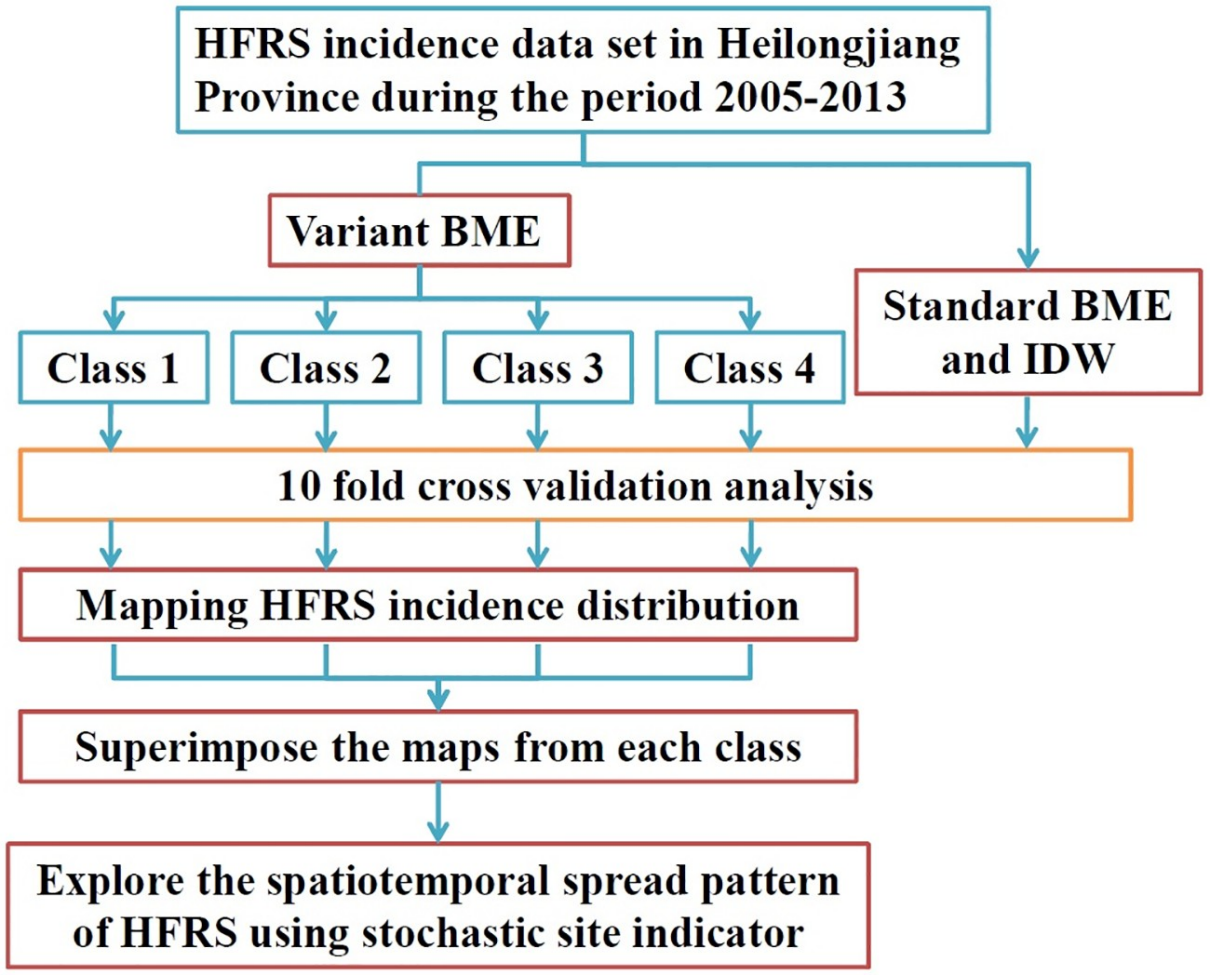
Outline of the cd-BME framework.

The spatiotemporal mapping of HFRS incidence distribution was performed using the BME method [21] by simultaneously assimilating the core or general knowledge base (*G*-KB) and the site-specific or specificatory knowledge base (*S*-KB) of HFRS in Heilongjiang Province. Specifically, the mean and covariance functions were treated as *G*-KB and the log-transformed HFRS data *Y*(***p***) was regarded as *S*-KB. The implementation of the BME method (S4 Text) to separately analyze data classes defined in terms of percentiles (Table 1) will be termed class-dependent BME (cd-BME). The cd-BME mapping results will be subsequently superimposed and back-transformed to obtain the final HFRS incidence space-time maps, which provide information about the actual form of the spatiotemporal HFRS spread. For comparison purposes, the standard BME implementation and the mainstream inverse distance weighting (IDW) method were also employed to analyze the original data set without class-decomposition. To evaluate the performance of the different approaches, a ten-fold cross validation analysis was conducted that involved three distinct accuracy indicators: the root mean square error (RMSE), the mean absolute error (MAE), the determination coefficient of the corresponding linear regression (*R*^2^). Space-time computational data analysis (BME) used the software library Spatiotemporal Epistemic Knowledge Synthesis-Graphical User Interface (SEKS-GUI, [30]), while the IDW was implemented using R software [31]. An outline of the proposed methodological framework is presented in Fig 2.

### Stochastic indicators of space-time HFRS dependency

The spatiotemporal arrangement of the HFRS patches occupied by the different incidence classes can be described by the relative frequencies with which the different kinds of incidence classes occur next to one another, i.e., by the corresponding incidence probabilities across space and time. That is to say, the spatiotemporal dependence of HFRS incidences can be assessed quantitatively in terms of stochastic (probabilistic) indicators linking categorical HFRS incidences at points ***p*** and ***p***′ in various yet complementary ways. Similar stochastic indicators have been used to characterize the space-time variation of population health status, environmental pollution and ocean health (e.g., [32–38]). The stochastic HFRS indicators considered in this work are based on the probability logic theory of medical reasoning developed in [29] and provide intuitive measures of relatedness or logical correlations between two categorical HFRS incidences at points ***p*** and ***p***′, and they may be estimated along multiple directions (anisotropic relatedness) or omnidirectionally (isotropic relatedness). Specifically:

#### The joint incidence probability

The joint incidence probability (JIP) is an extension in the stochastic (probabilistic) domain of the joint categorical incidence occurrence *X*(***p***) ∈ *I*_*m*_ ∧ *X*(***p***′) ∈ *I*_*n*_ at the space-time points ***p*** and ***p***′ (the symbol “∧” means “and”), with truth table shown in the first three columns of Table 2, where the *T* and *F* denote true (i.e., *X*(***p***) ∈ *I*_*m*_ or *I*_*n*_) and false (i.e., *X*(***p***) ∉ *I*_*m*_ or *I*_*n*_), respectively. The JIP measures the probability that at the points ***p*** and ***p***′ the HFRS incidences belong to the specified class intervals *I*_*m*_ and *I*_*n*_, respectively, i.e., the probability that the incidences *X*(***p***) ∈ *I*_*m*_ and *X*(***p***′) ∈ *I*_*n*_ occur simultaneously, so that

$$JIP_{X}^{m,n}(p,p^{\prime })=P[X(p)\in {Ι}_{m}\wedge X(p^{\prime })\in {Ι}_{n}].$$ (1)

**Table 2:** Truth table of the joint incidence, the incidence implication and the incidence equivalence.

*X*(*p*) ∈ *I*_*m*_	*X*(*p*′) ∈ *I*_*n*_	*X*(*p*) ∈ *I*_*m*_ ∧ *X*(*p*′) ∈ *I*_*n*_	*X*(*p*) ∈ *I*_*m*_ → *X*(*p*′) ∈ *I*_*n*_	*X*(*p*) ∈ *I*_*m*_ ↔ *X*(*p*′) ∈ *I*_*n*_
*T*	*T*	*T*	*T*	*T*
*T*	*F*	*F*	*F*	*F*
*F*	*T*	*F*	*T*	*F*
*F*	*F*	*F*	*T*	*T*

Otherwise said, the JIP calculates the connectivity (strength of dependency) between the HFRS incidences *X*(***p***) ∈ *I*_*m*_ and *X*(***p***′) ∈ *I*_*n*_ in the space-time domain of interest. The higher the JIP is, the stronger the connectivity between incidences belonging to the interval classes *I*_*m*_ and *I*_*n*_ (or, equivalently, the higher the probability of HFRS spreading from class *m* to class *n*).

#### The incidence implication probability

The incidence implication probability (IIP) is an extension in the probabilistic domain of the standard logical implication *X*(***p***) ∈ *I*_*m*_ → *X*(***p***′) ∈ *I*_*n*_ (also known as material conditional) with truth table also shown in Table 2. In this case, the IIP probability can be written as [39],

$$IIP_{X}^{m,n}(p,p^{\prime })=P[X(p)\in I_{m}\to X(p^{\prime })\in I_{n}],$$ (2)

which expresses the strength of the logical implication *X*(***p***) ∈ *I*_*m*_ → *X*(***p***′) ∈ *I*_*n*_, i.e., that the categorical incidence *X*(***p***) ∈ *I*_*m*_ implies logically the categorical incidence *X*(***p***′) ∈ *I*_*n*_. Sometimes the class *I*_*m*_ is called the antecedent class, and *I*_*n*_ the consequent class. The *I*_*m*_ and *I*_*n*_ in IIP are not interchangeable, due to IIP’s asymmetry. From Table 2 we observe that the logical implication holds when (*X*(***p***) ∈ *I*_*m*_ ∧ *X*(***p***′) ∈ *I*_*n*_), i.e., when it is not the case that both incidences *X*(***p***) ∈ *I*_*m*_ and *X*(***p***′) ∈ *I*_*n*_ occur. As such, IIP measures the probability that *X*(***p***) ∈ *I*_*m*_ and *X*(***p***′) ∉ *I*_*n*_ do not occur simultaneously across the Heilongjiang province during 2005–2013. Equivalently, the IIP measures the probability that either the categorical incidence *X*(***p***) ∈ *I*_*m*_ or the incidence *X*(***p***′) ∈ *I*_*n*_ occurs. Obviously, the larger the IIP is, the stronger the spatiotemporal dependence of the HFRS incidence distribution.

#### The equivalency incidence probability

The equivalency incidence probability (EIP) is an extension in the probabilistic domain of the standard logical equivalence *X*(***p***) ∈ *I*_*m*_ ↔ *X*(***p***′) ∈ *I*_*n*_ (also known as logical biconditional), with truth table as shown in Table 2. In this case, the IIP probability can be written as,

$$EIP_{X}^{m,n}(p,p^{\prime })=P[X(p)\in I_{m}↔X(p^{\prime })\in I_{n}],$$ (3)

which expresses the strength of the logical equivalence *X*(***p***) ∈ *I*_*m*_ ↔ *X*(***p***′) ∈ *I*_*n*_, i.e., it calculates the probability that the categorical incidence *X*(***p***) ∈ *I*_*m*_ is logically equivalent to the incidence *X*(***p***′) ∈ *I*_*n*_. From Table 2 we observe that the logical equivalence holds when both or neither of *X*(***p***) ∈ *I*_*m*_ and *X*(***p***′) ∈ *I*_*n*_ occur simultaneously, i.e.,

$$\left(X(p)\in I_{m}\wedge X(p^{\prime })\in I_{n})\vee (X(p)\notin I_{m}\wedge X(p^{\prime })\notin I_{n}\right)$$

(the symbol “∨” means “either, or”). As such, the EIP measures the degree to which the categorical incidences *X*(***p***) ∈ *I*_*m*_ and *X*(***p***′) ∈ *I*_*n*_ either occur simultaneously or they do not occur simultaneously. The EIP indicates a closer spatiotemporal dependence of the HFRS incidence distribution across the Heilongjiang province during 2005–2013.

#### Links with statistical incidence conditional

The statistical incidence conditional (SIC) represents the ratio of the number of HFRS distributions in which the categorical incidences *X*(***p***) ∈ *I*_*m*_ and *X*(***p***′) ∈ *I*_*n*_ occur simultaneously over the number of HFRS distributions in which the incidence *X*(***p***) ∈ *I*_*m*_ occur, i.e.,

$$SIC_{X}^{m,n}(p,p^{\prime })=P[X(p^{\prime })\in I_{n}|X(p)\in I_{m}],$$ (4)

which is a conditional categorical incidence probability.

#### Indicator assumptions and features

If space-time homostationarity is assumed (i.e., the HFRS incidence distribution is space homogeneous and time stationary), the indicators are functions of *Δ****p*** = ***p*** − ***p***′ = (***s*** − ***s***′, *t* − *t*′) = (***h***, *τ*). For example, the JIP can be written as

$$JIP_{X}^{m,n}(p,p^{\prime })=JIP_{X}^{m,n}(\Delta p)=JIP_{X}^{m,n}(h,\tau )$$ (5)

for all ***p*** and ***p***′ such that *Δ****p*** = (***h***, *τ*). Isotropy further implies that the JIP is only a function of the length |***h***| and time separation *τ*, i.e., the JIP is a function of |*Δ****p***| = (|***h*|**, *τ*). Similar expressions can be derived for the other three indicators in the case of space-time homostationarity and/or isotropy.

In addition, the four stochastic (probabilistic) HFRS indicators above convey complementary information regarding the intraclass relationship between the same incidence class (i.e., *m* = *n*), and the interclass relationship between different incidence classes (i.e., *m* ≠ *n*) in Heilongjiang province during January 2005–December 2013 (in fact, intraclass incidence variations can be considerably different than interclass HFRS variations). These stochastic HFRS indicators and their interpretations are summarized in Table 3. Interestingly, the JIP, IIP and EIP indicators can be expressed in terms of the SIC (these relationships are displayed in the third column of Table 3). Further discussion of the characteristics and interpretations of the four stochastic indicators can be found in S5 Text. In practice, these interpretations can be used in a complementary manner to obtain a holistic assessment of the disease situation. Next, the above stochastic HFRS indicators are calculated using space-time maps of HFRS incidence generated by categorized (cd) BME and compared in the case of the Heilongjiang province that is the focus of this work.

**Table 3:** Stochastic HFRS indicators and their links to the statistical conditional.

*HFRS indicator*	*Probability that the categorical HFRS incidences*	*Relationship with statistical incidence conditional*
$JIP_{X}^{m,n}(p,p^{\prime })$	*X*(***p***) ∈ *I*_*m*_ and *X*(***p***′) ∈ *I*_*n*_ occur simultaneously	$SIC_{X}^{m,n}(p,p^{\prime })P[X(p)\in I_{m}]$
$IIP_{X}^{m,n}(p,p^{\prime })$	*X*(***p***) ∈ *I*_*m*_ and *X*(***p***′) ∈ *I*_*n*_ do not occur simultaneously	$SIC_{X}^{m,n}(p,p^{\prime })P[X(p)\in I_{m}]+P[X(p)\notin I_{m}]$
$EIP_{X}^{m,n}(p,p^{\prime })$	*X*(***p***) ∈ *I*_*m*_ and *X*(***p***′) ∈ *I*_*n*_ either occur simultaneously or do not occur simultaneously	$2SIC_{X}^{m,n}(p,p^{\prime })P[X(p)\in I_{m}]+P[X(p)\notin I_{m}]-P[X(p^{\prime })\in I_{n}]$
$SIC_{X}^{m,n}(p,p^{\prime })$	*X*(***p***′) ∈ *I*_*n*_ occurs given that *X*(***p***) ∈ *I*_*m*_ occurred	

## Results

In what follows, the categorical HFRS incidence representation will be considered in terms of the four selected incidence interval classes, *I*_*m*_, *m* = 1, 2, 3, 4. In the context of the categorical HFRS representation used in this work, two kinds of HFRS spread patterns were assumed for comparison purposes: outward HFRS spread that links a specific incidence class to the entire set of incidence classes, and inward HFRS spread that is concerned with incidence transition from the entire set of classes to a specific class.

### Space-time mapping of HFRS incidences using class-based BME

#### Spatiotemporal correlation (covariance model) of HFRS incidences

**Fig 3:**
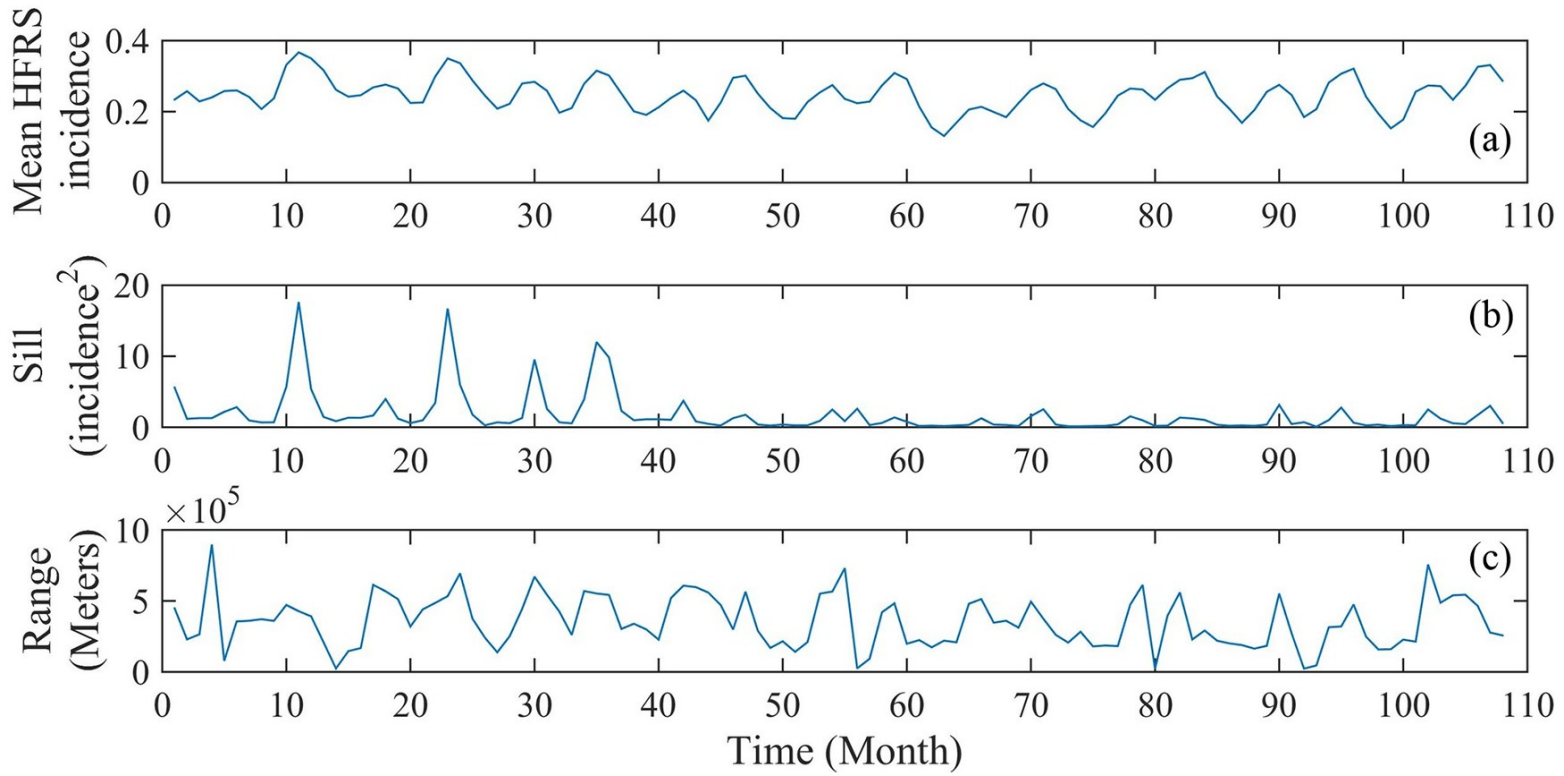
Monthly variation of (a) the HFRS mean, as well as (b) covariance sill and (c) range during 2005–2013.

**Fig 4:**
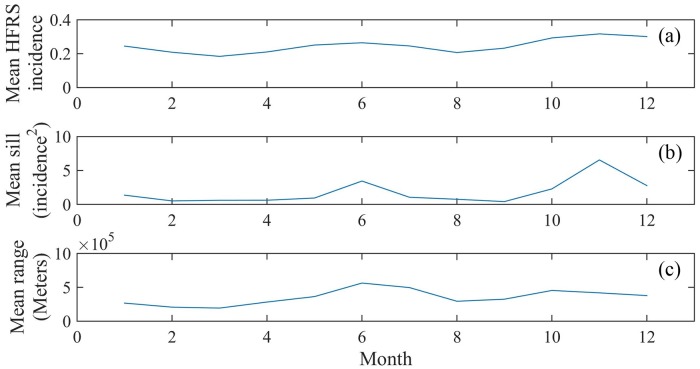
Plots of (a) the HFRS mean and covariance parameters, i.e., (b) sill and (c) range for the same month (January-December) averaged over the corresponding months of the period 2005–2013.

Monthly spatial correlations of the original HFRS incidence *X*(***p***) were first calculated (108 covariances, in total). The temporal variation of the HSRF mean is smooth and periodic, fluctuating constantly around the 0.25 incidence value (Fig 3a). The covariance sill values explain the variance of HFRS incidence in the Heilongjiang Province during the same month, exhibiting certain noticeable peaks at twelve months period, the size of which reduce with time (Fig 3b). The interpretation of these high peaks is that they imply the presence of high uncertainty (or the presence of outbreaks at specific locations) in the HFRS variation during the peak times. The correlation ranges determine the HFRS domain of influence, showing a rough variation with time, which, like the HFRS mean variation, it also exhibits a periodic character (Fig 3c). The minimum and maximum values of the correlation range during the period 2005–2013 are 21,700 meters and 897,650 meters, respectively. The functional shapes of the HFRS mean, covariance sill and range are remarkably similar (e.g., in Fig 4 large (small) HSRF mean values are directly linked to long (short) correlation ranges). Interpretationally, the two distinct peaks of the HSRF mean plot during June and November, which coincide with the corresponding peaks of the mean sill plot, detect the HFRS outbreaks that occurred during these months.

**Fig 5:**
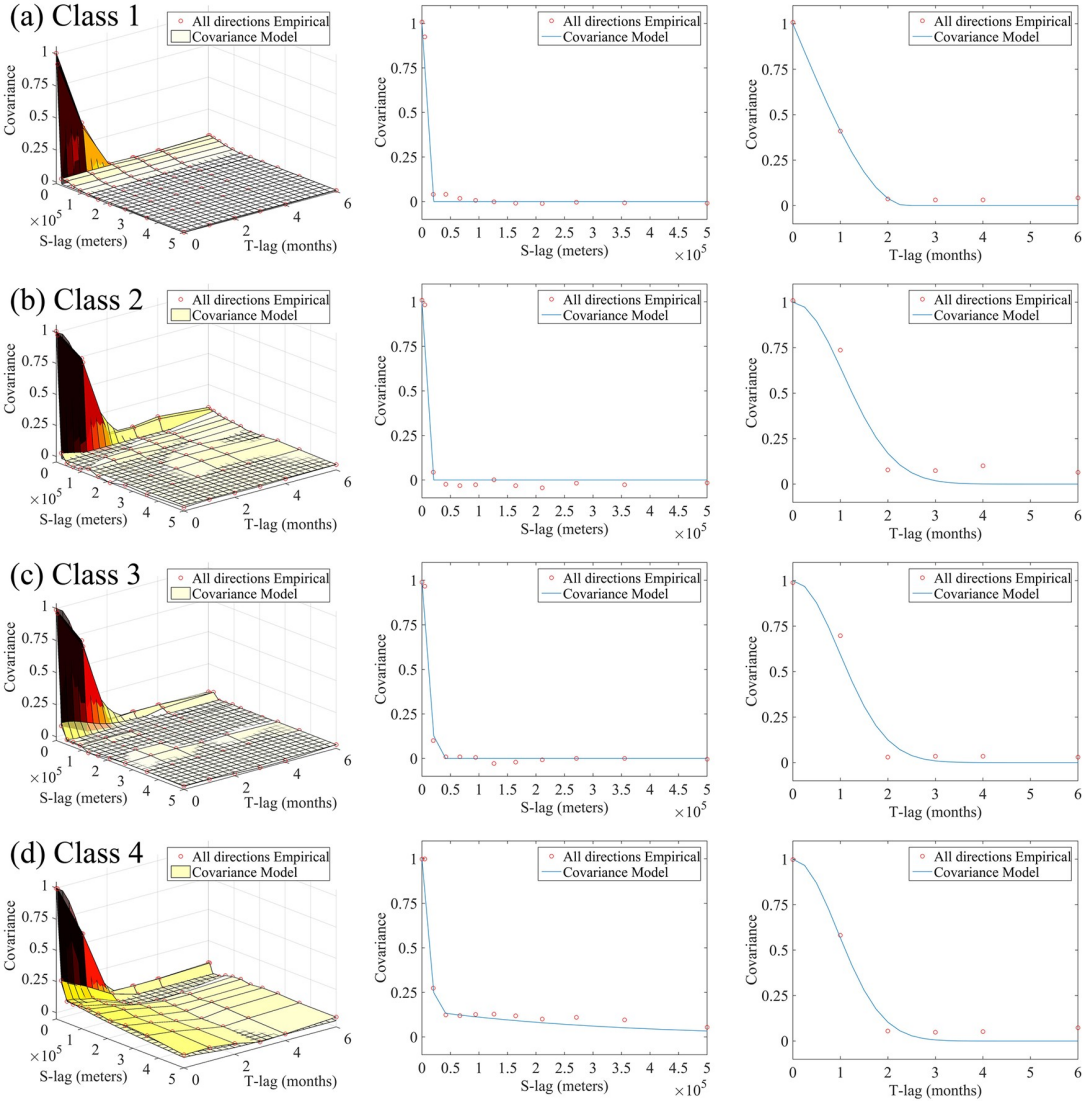
Plots of the empirical covariance values (red circles) and fitted theoretical models (multi-color surface or blue line in 3D, and 2D plots, respectively) of the log-transformed HFRS incidences (county level) for each incidence class, i.e., (a) for class 1, (b) for class 2, (c) for class 3 and (d) for class 4.

Subsequently, the empirical space-time covariance values and the fitted theoretical models of the log-transform HFRS incidences *Y*(***p***) (shown in Fig 5) were derived separately for each interval class of Table 1. It is noteworthy that the space-time dependence ranges of HFRS incidences vary with the incidence level (S2 Table). Moreover, all theoretical covariance models, *c*_*X*_(*Δ****p***) = *c*_*X*_(|***h***|, *τ*), are space-time separable. However, the spatial part of the covariance model in class *I*_4_ (corresponding to the highest incidence level) is more complicated than the other three, further indicating the different space-time variation pattern of class *I*_4_.

Some key space-time variation features of the empirical covariance values (red circles) and the corresponding theoretical models (lines) can be observed in Fig 5:

1. For all HFRS incidence classes considered, the non-zero slopes of the spatial covariance components at the origin indicate that the log-transformed HFRS incidence fields exhibit intense localized variations across space. Regarding the temporal HFRS covariance components, except for the incidence class 1, the slopes of these covariances at the origin are zero, implying (based on the underlying spatiotemporal random field theory) that the temporal incidence variation is apparently much smoother than the spatial incidence variation.
2. Visual inspection of the temporal and spatial covariance plots of the HFRS incidence in the Heilongjiang province, obtained for different incidence classes, reveals that the spatiotemporal structure of incidence distributions embodied in the shape of the space-time covariance functions depends on the HFRS incidence level, with dependencies in time being stronger than those in space.
3. The spatial and temporal covariance lags beyond which HFRS incidence dependencies are negligible (usually referred to as spatial and temporal correlation ranges, respectively) vary among the different incidence classes selected.
4. Beyond the corresponding spatial and temporal ranges, the spatial and temporal components of the covariance functions of the four HFRS incidence classes are approximately zero-valued, indicating that the log-transformed incidence is spatially homogeneous in space and stationary in time. Also, the selected theoretical models (see S2 Table of the SI section) provide good fits to the empirical covariance values, thus validating the adequate theoretical representation of the actual HFRS variation in Heilongjiang province.

#### Accuracy performance of cd-BME mapping

The ten-fold cross validation analysis shows that the cd-BME outperforms the standard (class-independent) BME and IDW techniques in terms of the corresponding RMSE, MAE and *R*^2^ values calculated using the entire HFRS incidence dataset (S3 Table). Moreover, in order to test the robustness of the cd-BME, the original data set was also divided into 3 classes (with percentile ranges 0–72%, 72–86% and 86–100%), in which case the 10 fold-cross validation results showed that the cd-BME with 3 classes also outperforms the standard BME and IDW, although it is inferior to the cd-BME with 4 classes selected in this work (see, optimal selection of number of classes discussed in S3 Text). By looking into each individual HFRS interval class, the performance of both ways of BME implementation (cd and standard) decreases with increasing incidence level (i.e., from incidence class 1 to class 4) but, in all cases, the performance of the cd-BME implementation remains superior (S4 Table). In terms of the MAE indicator, the accuracy improvement when using the cd-BME implementation instead of the standard BME implementation is 65.16%, 82.13%, 72.32% and 42.18% for the four incidence classes considered, respectively.

#### Space-time mapping of HFRS incidences

The 108 spatiotemporal HFRS distribution maps with resolution 5 *km* × 5 *km* × 1 *month* were obtained for each interval class (i.e., 108 × 4 = 432 maps were generated, in total), and subsequently superimposed to produce the final HFRS distribution maps (S2–S10 Figs). For illustration purposes, S5 Fig shows the selected HFRS incidence map for the year 2008. One observes the following: (*a*) The HFRS incidences in Heilongjiang province exhibited two noticeable peaks during the months of June and November. (*b*) The first peak triggers the rapid spread of HFRS cases during the August-November period toward the western part of Heilongjiang province and also toward some counties in the eastern part of the province. (*c*) After November, the HFRS incidences decrease at some counties, and the number of counties suffering high levels of HFRS infections also decreases. (*d*) The western part of the Heilongjiang province exhibits a lower number of HFRS incidences than the eastern part.

**Fig 6:**
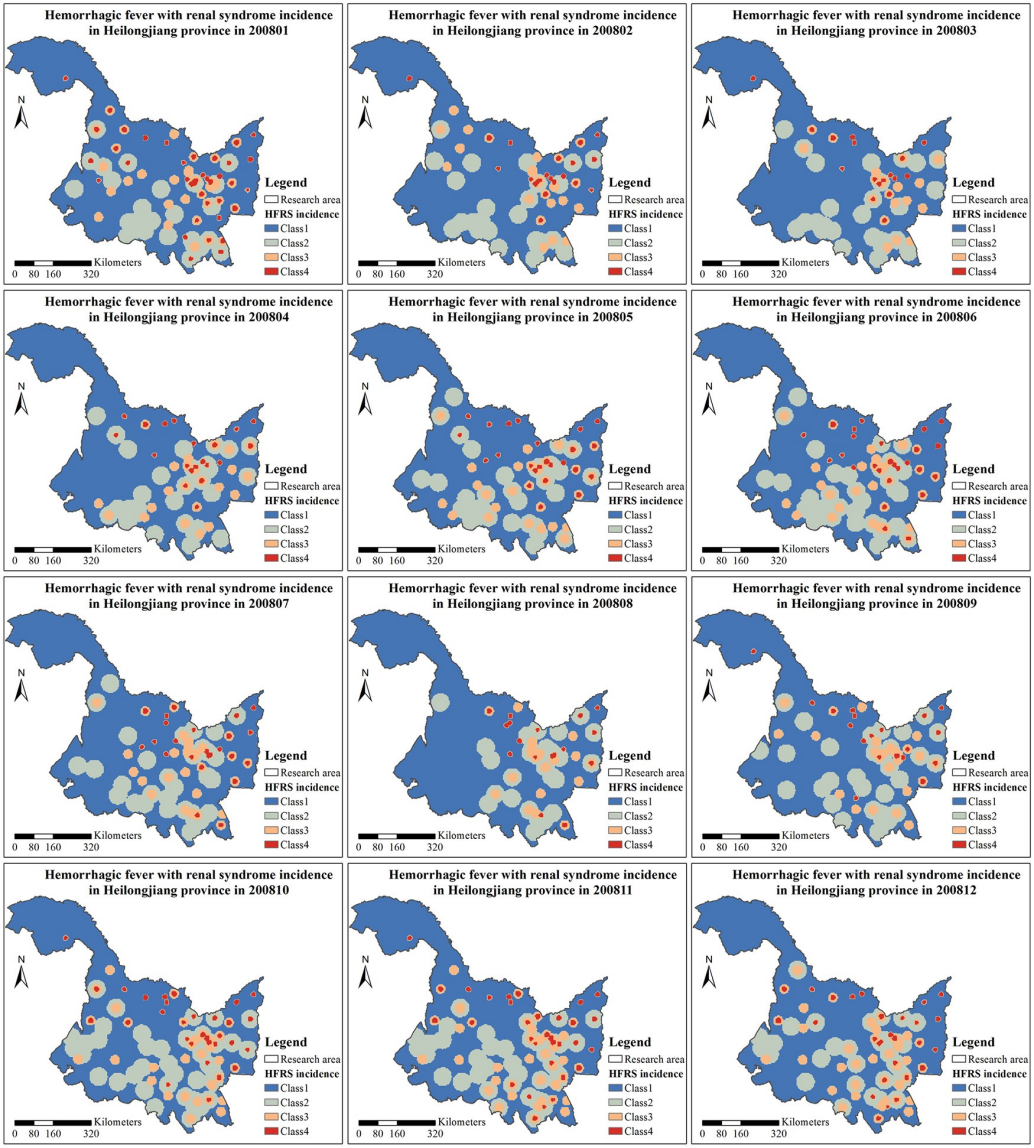
HFRS incidence maps of Heilongjiang province during 2008 in terms of the incidence classes considered.

Alternatively, an informative visualization of HFRS spread over the Heilongjiang province during 2008 is provided by the categorical space-time maps in terms of the four different incidence interval classes defined earlier, and shown in Fig 6. Specifically, the maps of Fig 6 present to scale the monthly distribution of “patches” with incidence classes *I*_2_, *I*_3_ and *I*_4_ amidst class *I*_1_. An apparent feature of Fig 6 is that some parts of the Heilongjiang region are dominated by small patches of HFRS incidence, whereas in some other parts the HFRS patches seem to be large. These incidence patches are mutually exclusive, bounded and they have varying sizes. Certain HFRS patches seem to be distinctly elongated toward some preferred direction, indicating the presence of anisotropy. Sometimes the HFRS patches are clearly separated from one another, some other times they share common boundaries, and yet some other times they are completely surrounded by another class (e.g., in various monthly maps the classes *I*_2_, *I*_3_ and *I*_4_ occur as patches within the class *I*_1_). The proportions of regional cover by each incidence class shows the dominance of class *I*_1_ within which all the other classes are embedded, so that class *I*_1_ acts like a background class.

In this work we also calculated the mean distances across patches during each month of the period 2005–2013 using the centers of each incidence patch. Given that the area of class *I*_1_ in Fig 6 is the background domain that is not consisting of patches, a relevant center of class *I*_1_ cannot be recognized in Fig 6, and, hence, mean distances could not be calculated for class *I*_1_. The plots of mean distances across patches during each month of the period 2005–2013 are shown in S11 Fig (e.g., the mean distance between the incidence class *I*_2_ and the class *I*_3_ during March 2008 is 258.3 *Km*). These mean distances across patches may be interpreted as effective ranges beyond which the transition probabilities remain essentially constant. As we observe in S11 Fig, the temporal variations of the mean distances across patches are stationary, i.e., they fluctuate around certain constant values, as follow: 272 *Km* (mean distance across *I*_2_ patches), 304 *Km* (across *I*_2_ − *I*_3_ patches), 340 *Km* (across *I*_2_ − *I*_4_ patches), 255 *Km* (across *I*_3_ patches), 273 *Km* (across *I*_3_ − *I*_4_ patches), and 273 *Km* (across *I*_4_ patches). I.e., the longest mean distance during 2005–2013 occurred across patches of class *I*_2_ to *I*_4_, and the shortest mean distance across patches of class *I*_2_. The reciprocals of the mean distances between incidence patches can also serve as the intensity parameters of the four classes. As the numerical results above demonstrate, nevertheless, there are small differences among the intensity parameters of the four HFRS classes, with the relatively most intense being the *I*_3_ patches (4 × 10^−4^ Km^-1^), followed by the *I*_2_, *I*_4_ and *I*_3_ − *I*_4_ patches (3.6 × 10^−4^ *Km*^-1^), and with the least intense being the *I*_2_ − *I*_3_ (3.3 × 10^−4^ *Km*^-1^) and *I*_2_ − *I*_4_ (2.9 × 10^−4^ *Km*^-1^) patches. The mean coefficients of variation of the HFRS incidence during the period 2005–2013 were equal to 57.6532, 0.0232, 0.0824 and 0.2997 for the *I*_1_, *I*_2_, *I*_3_ and *I*_4_ class, respectively.

It would be interesting to calculate the varying frequencies and dynamics of adjacent and non-adjacent HFRS classes in the Heilongjiang province during 2005–2013, as well as the probabilities of incidence transition from one class to another. This is the concern of the stochastic (probabilistic) HFRS indicators to be discussed next.

### Spatiotemporal dependence pattern of HFRS incidence spread in Heilongjiang province during 2005–2013

The global size of each incidence class during Jan 2005-Dec 2013 was first calculated (S6 Text). At time *t* let $\bar{X}(t)$ denote the spatial mean of the HFRS incidence *X*(***s***, *t*) averaged over the Heilongjiang region. For each time *t* (month), the probability $P[\bar{X}(t)\in I_{m}]$ represents the geographical fraction of the Heilongjiang region with $\bar{X}(t)\in I_{m}$ (*m* = 1, 2, 3, 4).S12a–S12d Fig present the temporal variation of $P[\bar{X}(t)\in I_{m}]$ for each of the four HFRS classes. The ${\bar{JIP}}_{X}^{m}(t)$ values for *m* = 1, 2, 3 and 4 fluctuate around the constant probability values, ℘_1_ = 0.6767, ℘_2_ = 0.2314, ℘_3_ = 0.0736, ℘_4_ = 0.0184, respectively. Clearly, the HFRS class *I*_1_ is the most dominant in the Heilongjiang province (covering about 68% of the total area), followed by the classes *I*_2_, *I*_3_ and *I*_4_.

The spatiotemporal arrangement of the different HFRS incidence classes relative to each other and their relationships were assessed based on the different perspectives offered by the four stochastic indicators considered in this work (their detailed calculation can be found in S7 Text). The obtained results are presented next.

#### The JIP perspective

**Fig 7:**
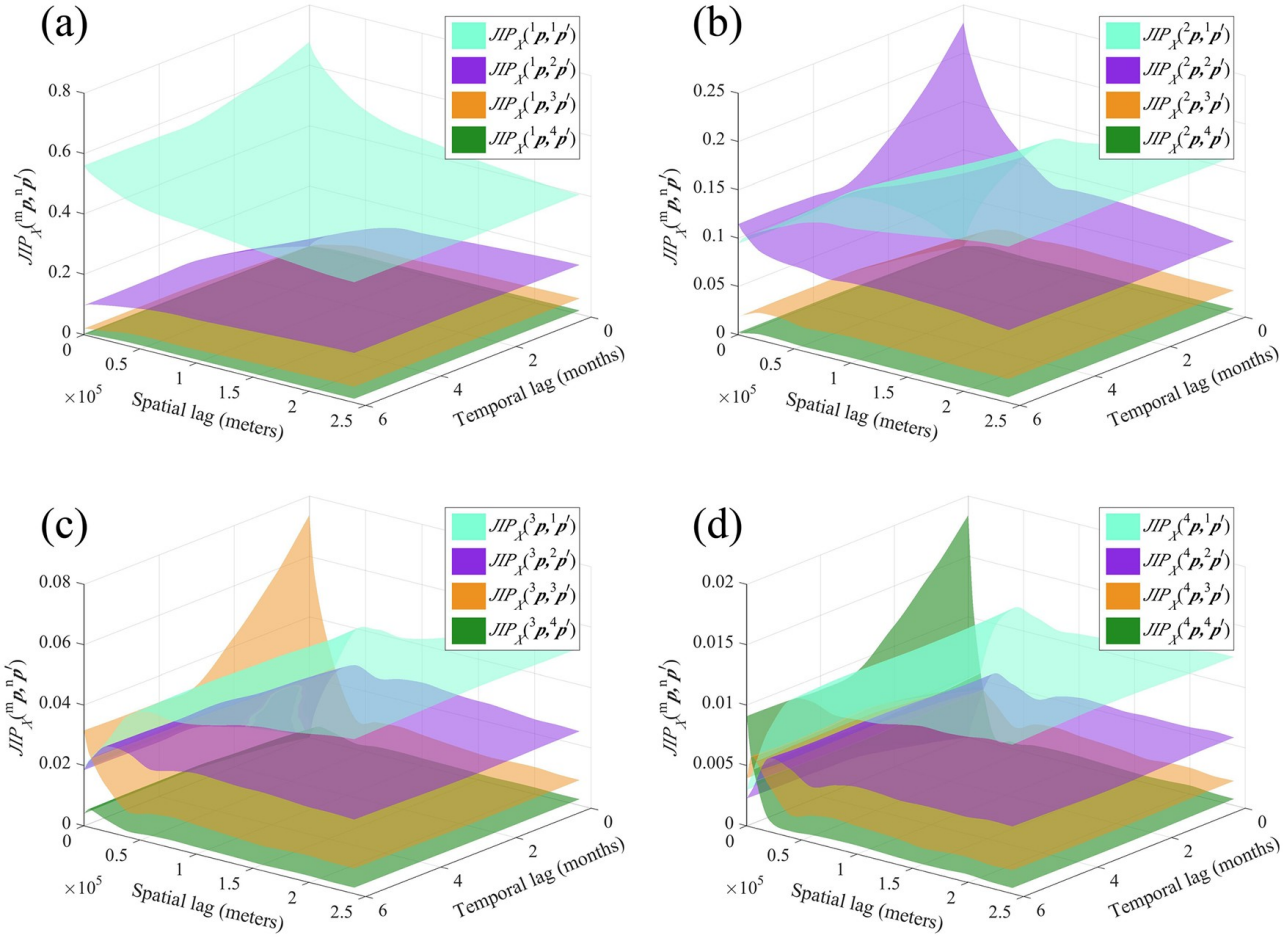
The *JIP*_*X*_(^*m*^*p*, ^*n*^*p*′) plots in Heilongjiang province when (a) *m* = 1, (b) *m* = 2, (c) *m* = 3 and (d) *m* = 4.

Assuming that the space-time points ***p*** and ***p*′** were randomly selected in the study domain, the $JIP_{X}^{m,n}(p,p^{\prime })$ surfaces of Fig 7 express the joint probability of occurrence of both HFRS incidences *X*(***p***) ∈ *I*_*m*_ and *X*(***p***′) ∈ *I*_*n*_ as a function of the spatial distance ***h*** between these two points and their time separation *τ*. Otherwise said, given that *τ* = *t*′ − *t* > 0, the JIP value is equal to the probability that the HFRS incidence moves from an incidence patch of class *I*_*m*_ at ***p*** to a patch of class *I*_*n*_ at ***p***′.

We observe that the $JIP_{X}^{m,m}(h,\tau )$ surfaces in Fig 7 representing the distribution of intraclass HFRS dependence (i.e., *m* = *n*) start from their highest value at ***h*** = 0, *τ* = 0, and gradually decrease to a stable, for all practical purposes value. At large space-time lags, this value should be equal to ${℘}_{m}^{2}$. The lag ***h*** and separation *τ* corresponding to this stable value defines the spatial and temporal ranges representing the distance and time of self-dependence of the *I*_*m*_ class. The height of the JIP surfaces also drops gradually from class *I*_1_ to class *I*_4_, i.e., the space-time dependence is higher among smaller incidences than among larger ones, and the incidence class *I*_1_ has the most significant contribution to the strength of the space-time HFRS dependence measured by JIP compared to the other three classes. In fact, the probability between categorical incidences of class *I*_1_ is higher than between incidences in any other class, even at long ***h*** and *τ* (compare the cyan surfaces in Fig 7a with the purple, orange and green surfaces in Fig 7b, 7c and 7d, respectively). Similarly, for classes *I*_1_ and *I*_2_, there is a higher probability of decreasing intraclass HFRS dependence with increasing ***h*** and *τ* (Fig 7a and 7b), whereas for classes *I*_3_ and *I*_4_ the intraclass HFRS dependence is the strongest at small temporal lags *τ* (Fig 7c and 7d).

As regards interclass variation (i.e., *m* ≠ *n*), the JIP measures the probability of moving from an incidence patch of class *I*_*m*_ to one of class *I*_*n*_. The interclass JIP plots represent the change in transition probabilities between two different classes from one point to another point with increasing ***h*** and *τ*. The JIP probability surfaces representing the space-time distribution of interclass HFRS dependence are higher between neighboring classes, i.e., the JIP surface of any incidence classes *m* and *n* drops with increasing difference |*n* − *m*| (e.g., the JIP surface of class pair *I*_1_ and *I*_2_ is higher than that of the pair *I*_1_ and *I*_3_, which, in turn is higher than that of the pair *I*_1_ and *I*_4_). The probability of HFRS dependence between class *I*_1_ and the other four classes decreases with increasing HFRS incidence in class *I*_1_. Also, when either *X*(***p***) ∈ *I*_4_ or *X*(***p***′) ∈ *I*_4_ occur, the corresponding joint incidence probabilities $JIP_{X}^{4,n}(p,p^{\prime })$ or $JIP_{X}^{m,4}(p,p^{\prime })$ are the smallest among the $JIP_{X}^{m,n}(p,p^{\prime })$ values with *m*, *n* = 1, 2, 3 or 4 (see the green surface in Fig 7a–7c and the four other colors surfaces in Fig 7d). Visually, class *I*_1_ exhibits the simplest space-time patterns of HFRS dependency among all four incidence classes considered, since the JIP surfaces in Fig 7a do not overlap. Lastly, the HFRS incidences of class *I*_4_ exhibit the lowest connectivity with the other incidence classes, as indicated by the lower JIP values in Fig 10d relative to the other classes in Fig 7a–7c), followed by class *I*_3_. (More characteristics of JIP values can be found in S8 Text).

#### The IIP perspective

**Fig 8:**
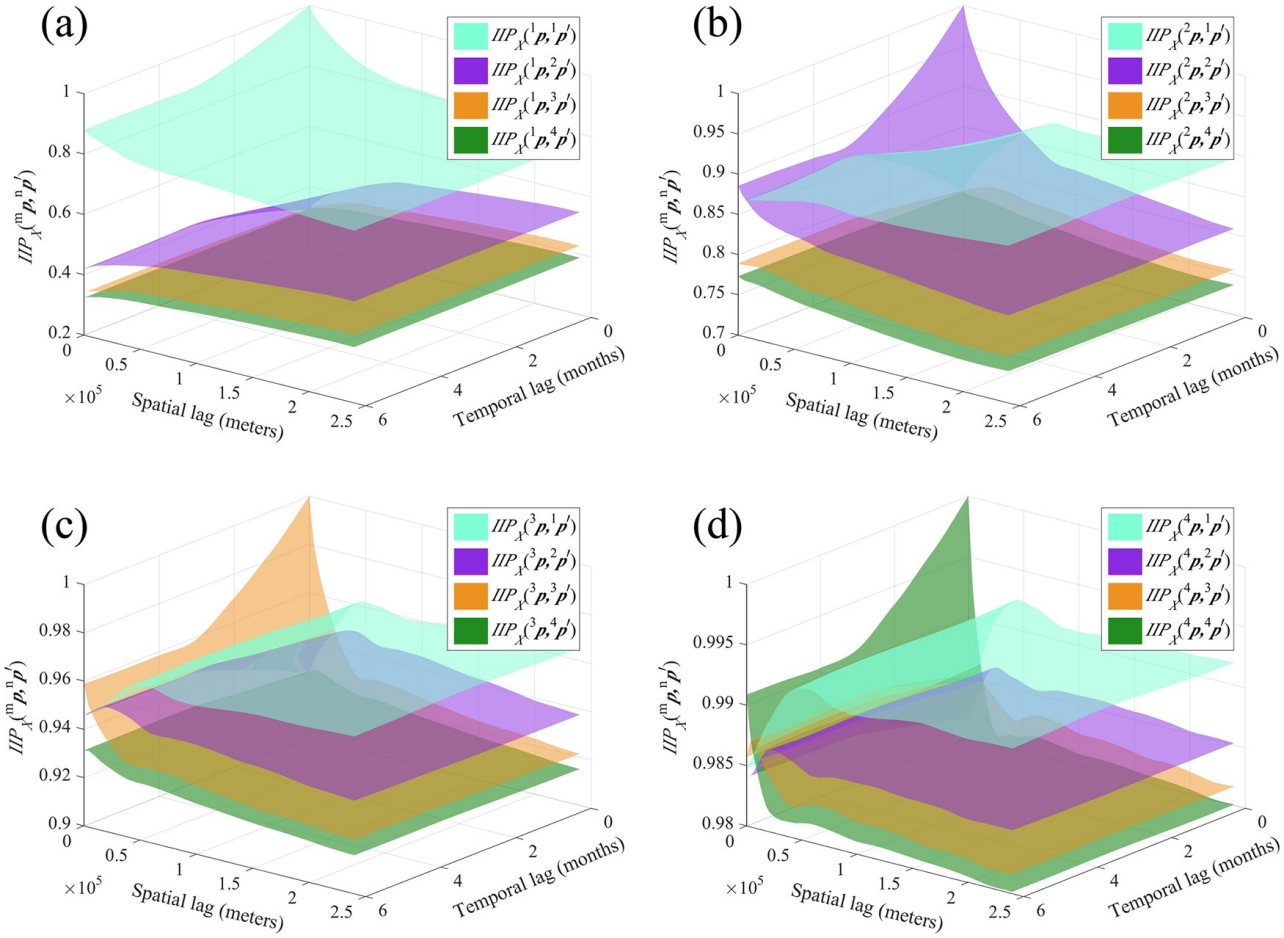
The *IIP*_*X*_(^*m*^*p*, ^*n*^*p*′) plots in Heilongjiang province when (a) *m* = 1, (b) *m* = 2, (c) *m* = 3 and (d) *m* = 4.

Fig 8 shows the IIP probability of *X*(***p***) ∈ *I*_*m*_ → *X*(***p***′) ∈ *I*_*n*_ as a function of the distance lag ***h*** and the time separation *τ*.

The intraclass incidence surfaces $IIP_{X}^{m,m}(h,\tau )$ represent the change in probabilities of an incidence class *I*_*m*_ from one space-time point to another point with increasing lag ***h*** and separation *τ*. At large space-time lags, the $IIP_{X}^{m,m}(h,\tau )$ should be equal to 1 + ℘_*m*_(℘_*m*_ − 1). More specifically, Fig 8a shows that, overall, among the four classes considered the highest IIP probability of intraclass HFRS incidence dependence at distance and time lags ***h*** and *τ* occurs between incidences of the same class *I*_1_, i.e., $IIP_{X}^{1,1}(h,\tau )$; whereas the lowest IIP probability occurs between incidences of classes *I*_1_ and *I*_4_, i.e., $IIP_{X}^{1,4}(h,\tau )$. These results confirm that for all ***h*** and *τ*, the influence of the categorical HFRS incidence at location ***s*** and time *t* on the incidence at location ***s*** + ***h*** and time *t* + *τ* is much stronger for class *I*_1_ than for class *I*_4_. Fig 8b shows that at small ***h*** and *τ* the incidence of class *I*_2_ at location ***s*** and time *t* has a higher probability of influencing incidence of the same class *I*_2_ at location ***s*** + ***h*** and time *t* + *τ* than of the other three classes. But, at larger ***h*** and *τ* the HFRS incidence of class *I*_2_ at location ***s*** and time *t* has higher probability of influencing the incidence of class *I*_1_ at location ***s*** + ***h*** and time *t* + *τ* than the incidence of the other three classes. Similar conclusions can be drawn from Fig 8c and 8d.

#### The EIP perspective

**Fig 9:**
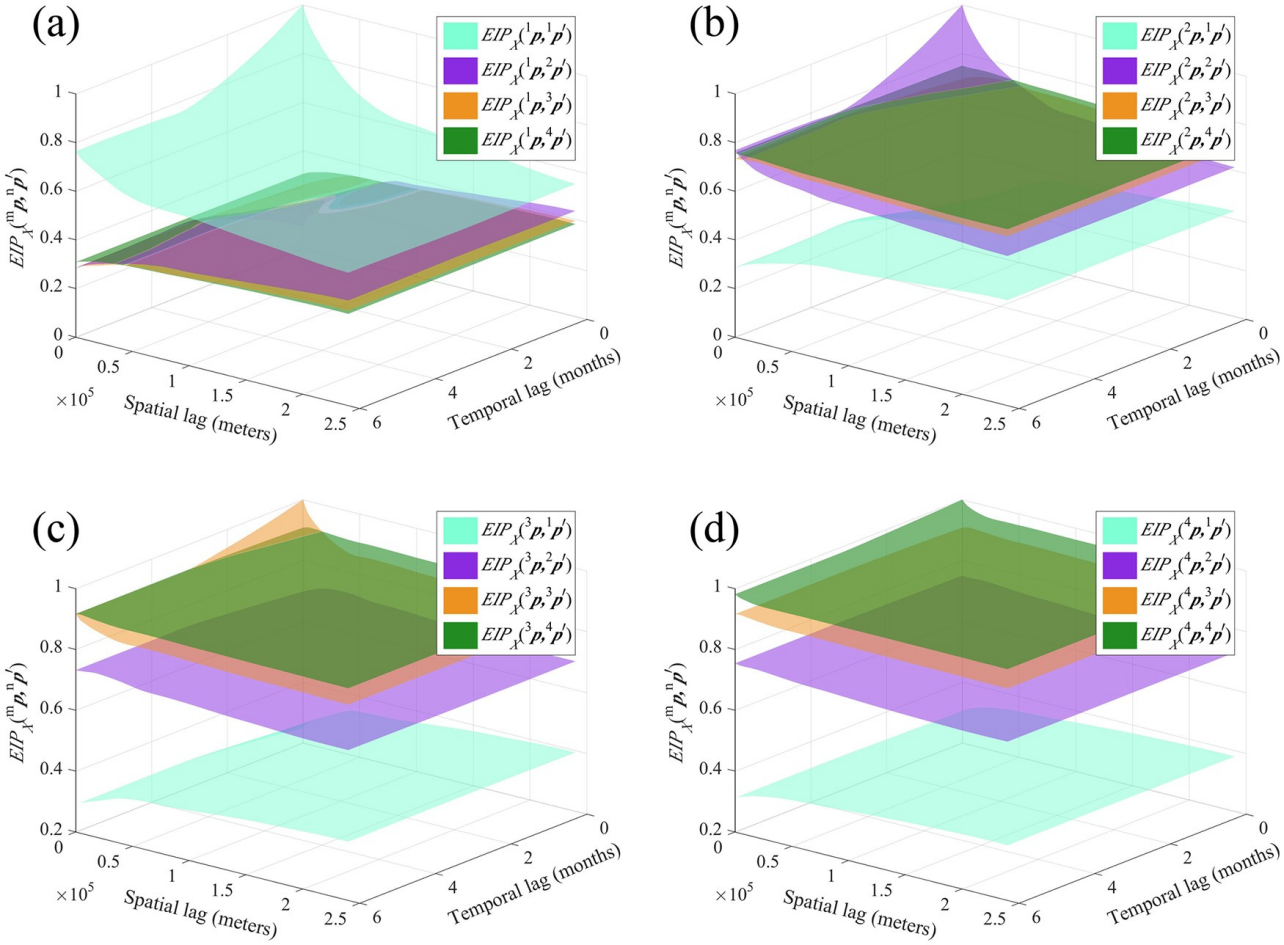
The *EIP*_*X*_(^*m*^*p*, ^*n*^*p*′) plots in Heilongjiang province when (a) *m* = 1, (b) *m* = 2, (c) *m* = 3 and (d) *m* = 4.

Fig 9 shows the probability surfaces representing the event that the categorical incidence *X*(***p***) ∈ *I*_*m*_ is logically equivalent to the incidence *X*(***p***′) ∈ *I*_*n*_, i.e., *X*(***p***) ∈ *I*_*m*_ ↔ *X*(***p***′) ∈ *I*_*n*_. In general, it holds that $EIP_{X}^{m,n}(p,p^{\prime })=EIP_{X}^{n,m}(p^{\prime },p)$. The EIP plots are the only one among the four indicators in which the surfaces associated with the different classes clearly overlap (see, e.g., Fig 9a). Further, by comparing the four Fig 9a–9d, we found that *X*(***p***′) ∈ *I*_4_ exhibits a high EIP probability of logical equivalence with *X*(***p***) ∈ *I*_*m*_ for *m* = 2, 3, 4, which also confirms the low connectivity between *X*(***p***) ∈ *I*_1_ and *X*(***p***′) ∈ *I*_4_ detected by JIP and IIP.

#### The SIC perspective

**Fig 10:**
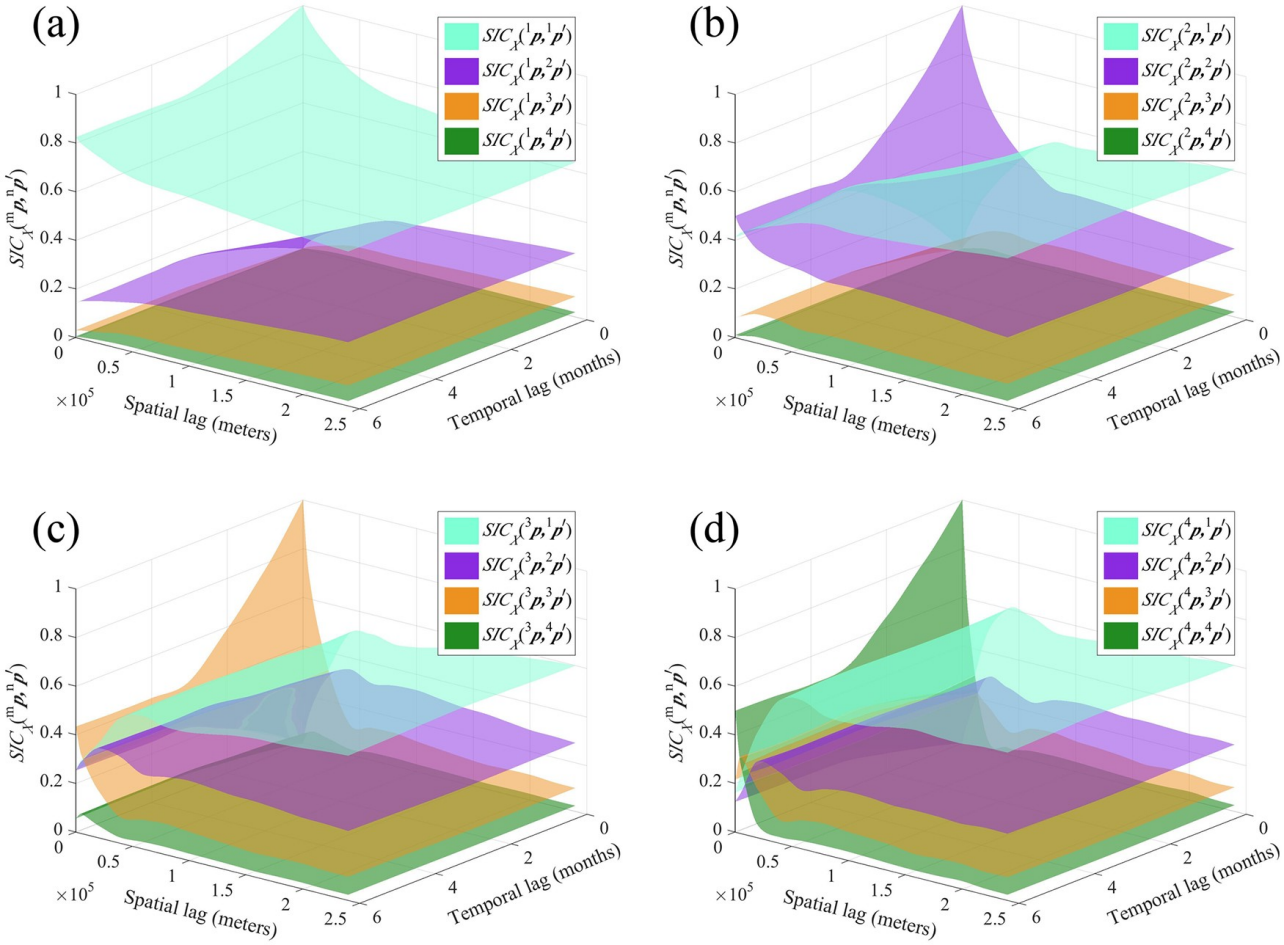
The *SIC*_*X*_(^*m*^*p*, ^*n*^*p*′) plots in Heilongjiang province when (a) *m* = 1, (b) *m* = 2, (c) *m* = 3 and (d) *m* = 4.

The SIC perspective focuses on the conditional probability that a patch of HFRS incidence class *I*_*n*_ lies next to one of species *I*_*m*_. Accordingly, Fig 10 shows the probability surfaces $SIC_{X}^{m,n}(p,p^{\prime })$, i.e., the probability of *X*(***p***′) ∈ *I*_*n*_ occurrence given that *X*(***p***) ∈ *I*_*m*_ occurred. Hence, SIC is the ratio of the number of HFRS distributions in which the categorical incidences *X*(***p***) ∈ *I*_*m*_ and *X*(***p***′) ∈ *I*_*n*_ occur simultaneously over the number of HFRS distributions in which the incidence *X*(***p***) ∈ *I*_*m*_ occur. The intraclass $SIC_{X}^{m,m}(p,p^{\prime })$ plots start from their highest value at ***h*** = 0, *τ* = 0, and gradually decrease to a stable, for all practical purposes, value, which, at large space-time lags is approximately equal to ℘_*m*_. On the other hand, the interclass $SIC_{X}^{m,n}(p,p^{\prime })$ plots start from their lowest value at ***h*** = 0, *τ* = 0, and then increase to a stable value, which, at large space-time lags is approximately equal to ℘_*n*_. The $SIC_{X}^{m,n}(p,p^{\prime })$ generally differs from $SIC_{X}^{m,n}(p^{\prime },p)$, the probability of *X*(***p***) ∈ *I*_*n*_ occurrence given that *X*(***p***′) ∈ *I*_*m*_ occurred, because ℘_*m*_ ≠ ℘_*n*_ (i.e., the classes *I*_*m*_ and *I*_*n*_ are not necessarily equiprobable).

Specifically, we observe that the SIC probability surfaces representing the space-time distribution of intraclass HFRS incidence dependence (i.e., *m* = *n*) drop gradually from class *I*_1_ to class *I*_4_. Moreover, the SIC values of intraclasses decrease with increasing ***h*** and *τ* (see the cyan, purple, brown and green surface in Fig 10a, 10b, 10c and 10d, respectively). Also, at large ***h*** and *τ* the SIC probability related to class *I*_1_ is always higher than that of the other three classes (see the cyan surfaces in Fig 10a–10d); whereas at small ***h*** and *τ* with *m* = *n*, the $SIC_{X}^{m,n}(p,p^{\prime })$ surface shows higher values than the $SIC_{X}^{m,n}(p,p^{\prime })$ surfaces with *m* ≠ *n*. Lastly, the SIC probability surfaces representing the space-time distribution of inter-class HFRS dependence (i.e., *m* ≠ *n*) are higher between neighboring classes, i.e., the SIC surface of any incidence classes *m* and *n* drops with increasing difference *n* − *m* ≥ 0 (e.g., the SIC surface of classes 1 and 2 is higher than that of classes 1 and 3, which, in turn is higher than that of classes 1 and 4, shown in Fig 10a). On the other hand, the SIC surface increases with increasing difference (*n* − *m* ≤ 0) at large ***h*** and *τ* (e.g., the $SIC_{X}^{3,2}(p,p^{\prime })$ is higher than $SIC_{X}^{3,3}(p,p^{\prime })$, whereas the $SIC_{X}^{3,1}(p,p^{\prime })$ is higher than $SIC_{X}^{3,2}(p,p^{\prime })$). The shapes of the SIC plots differ, depending on the spatiotemporal distribution of the *I*_*m*_ and *I*_*n*_ classes. For example, in Fig 10d the $SIC_{X}^{4,1}(p,p^{\prime })$ plot first exhibits certain peaks and then reaches its stable (asymptotic) value, which means that the incidence class *I*_1_ frequently occurs adjacent to classes *I*_4_. On the other hand, in Fig 10b the $SIC_{X}^{2,3}(p,p^{\prime })$ plot initially exhibits a low-value section and subsequently reaches its stable value, which means that the incidence class *I*_3_ seldom occurs close to the incidence class *I*_2_.

In theory it should hold that, *JIP*_*X*_ < *EIP*_*X*_ < *IIP*_*X*_, and *JIP*_*X*_ < *SIC*_*X*_, which are also confirmed in practice by the numerical results obtained in this study (S9 Text). Moreover, the space-time averaged values of the four stochastic HFRS indicators (i.e., *JIP*_*X*_, *IIP*_*X*_, *EIP*_*X*_, *SIC*_*X*_) lead to the following probability assessments (see, also, S10 Text):

- the probability of “both the interclass HFRS incidences occur at ***p*** and ***p***′” is very low (with probability 0.132),
- the probability of “either the HFRS incidence occurs at ***p***′ or it does not occur at ***p***” is very high (0.878),
- the probability of “either both HFRS incidences occur or both do not” is moderate (0.368); and
- the probability of “the HFRS incidence occurs at ***p***′ given that it occurs at ***p***” is relatively high (0.517).

The four different probabilities calculated above offer complementary quantitative assessments of the HFRS incidence dependency between any pair of points in the space-time domain of interest, with relative significance depending on the HFRS perspective adapted.

## Discussion

In this work, the spatiotemporal distribution of HFRS incidences in Heilongjiang province during the period 2005–2013 was studied quantitatively using spatiotemporal random field modeling that accounts for both the structural and uncertain aspects of HFRS spread. In this modeling context, key attributes of the HFRS incidence distribution were quantified by means of covariance functions, shedding light on the predominant HFRS spread patterns in space and time. A methodological advancement of the present study is that a cd-BME technique was introduced to reduce skewness effects in the spatiotemporal distribution of HFRS incidence data. This technique demonstrated a significant ability in modeling the pronounced variability in HFRS data caused by infection outbreaks that result in skewed distributions. Further, a moving windows configuration (Akita et al. [40]2012) was used that focuses mainly on local information (this configuration provides an HFRS variability representation that is closer to the real-world infectious disease spread pattern and less variable than the global pattern). The original dataset was optimally divided into four classes based on percentiles, which while maintained all advantages of BME modeling of spatiotemporal dependencies and correlations, it made HFRS predictions more accurate and robust.

In this work we considered several models, before concluding that BME is the best one for the Heilongjiang study. For example, due to the presence of disease data with a considerable number of 0 values, the zero-inflated model with spatial random effect was considered, because it has been designed to handle over-dispersed data [41]. We first noticed that after applying this model, the distribution of the original data will change and the data values will be also modified as a result. Second, this model usually either requires certain impact factors (e.g., environmental or gender population features) for regression purposes or the neighbor disease data should be assumed to be the independent variable (regressor). The former possibility is not applicable in the present study because of the lack of such data. As regards the latter possibility, taking the neighbor data as input could account to a limited extent for purely spatial dependency. Yet, these *ad hoc* approximations are not necessary here, since the BME method not only can handle data with any kind of distribution but it also rigorously accounts for the spatial, temporal and composite space-time dependency of the data. Other drawbacks of the zero-inflated model with spatial random effect is that it is a “naïve” interpolator (i.e., it does not account for the varying distances between data locations, and it is impossible to obtain informative space-time maps with finer resolution compared to the original dataset), and it cannot incorporate the disease spread indicators based on probability logic. BME has none of the above drawbacks (it is an adequate interpolator, i.e. it generates accurate estimates at unsampled locations that account for the varying distances between data locations and between the data and the interpolated locations, it generates informative space-time maps with fine resolution, and it incorporates the probabilistic disease indicators).

We concluded that the living conditions of rodents can dominate HFRS epidemics, as they influence the rodents-human interaction rate. As regards the space-time mapping of HFRS incidences in Heilongjiang Province during 2005–2013, the western part exhibits lower HFRS incidences than the eastern part. This is mainly due to environmental factors, as water resources expedite growth of plants, which serve as food and facilitate reproduction of rodents [42]. The aforementioned findings are in agreement with the broader picture of the river network in the region. More precisely, in Heilongjiang Province, only Neng River is located in the western part of Heilongjiang Province (i.e. lower HFRS incidences), whereas Wusuli River, Songhua River and Mudan River locate in the eastern part of the province (i.e. higher HFRS incidences) [15]. Moreover, the eastern and southeastern parts of Heilongjiang Province exhibit mixed land types with forests, favoring rodents’ reproduction. As a result, the eastern part of Heilongjiang Province demonstrates a consistently high level of HFRS infections throughout the year, whereas, the HFRS incidences in the western part of the province are dominated by monthly outbreaks. This is due to the fact that large proportions of the western part of the province are croplands, which remain flooded for crop growth purposes during the entire farming period, preventing rodents’ reproduction. A general overview of HFRS spread patterns in Heilongjiang Province can be obtained from Fig 5, as well as by the space-time maps in the supporting information (i.e. S2–S10 Figs).

Another contribution of this work is in filling the gap in the quantitative modeling of the spatiotemporal HFRS transition mechanisms based on probability logic. In other words, the present study provides a solution to the problem of calculating HFRS spread across space-time. In this context, the proposed stochastic HFRS indicators were applied to estimate the probability of HFSR transmission between space-time domains exhibiting different infection levels as a function their spatial distance and time separation. The results of the Heilongjiang study suggested that the proposed stochastic HFRS indicators summarize well the space-time incidence patterns, and their physical meanings and interpretations of the proposed HFRS indicators provide useful information about the HFRS spread mechanisms in the Heilongjiang province during Jan 2005-Dec 2013. Some HFRS classes were found to be cross-correlated with apparent correlation ranges, but some classes were not cross-correlated in the usual sense (i.e., if classes occur at two distant parts of the Heilongjiang province, it may be appropriate to characterize their interclass relationship as non-adjacent). Each HFRS class has a relationship with any other class in the Heilongjiang province during Jan 2005-Dec 2013, and quantifying the spatiotemporal relationships between HFRS classes and incorporating them into disease analysis and mapping are help us realistic assessment of the real HFRS situation in the space-time domain of interest. The transition probabilities provided by the HFRS indicators describe the spatiotemporal arrangements between incidence classes and suggest interactions that can be explored further in detail.

The plots of the four indicators offer complementary visualizations of the variation of the different probabilities of transition between incidence classes, i.e., the probabilities with which the different levels of HFRS incidences occur next to each other and so they describe the dependency pattern of the space-time arrangement of the HFRS patches occupied by the different incidence classes. Specifically, key features of interclass HFRS incidence relationships observed in this work are the space-time dependency, level of juxtaposition, and directional asymmetry of class patterns. The categorical HFRS incidences in the Heilongjiang province during Jan 2005-Dec 2013 exhibit rather smooth intraclass but complex interclass relationships. The complementary character of HFRS indicators of intraclass and interclass incidence transition and the estimated proportions of regional cover by each incidence class showed the dominance of the incidence class *I*_1_ with which all the other classes are associated. Indeed, the HFRS incidence class *I*_1_ covered about 68% of the total area, wheres the fact that the transition probabilities from the classes *I*_2_, *I*_3_ and *I*_4_ individually to class *I*_1_ are high suggests that the incidence class *I*_1_ dominated the Heilongjiang region. This phenomenon indicated that, independently of the particular infection level, the HFRS spread is dominated by point outbreaks with relatively small spatial ranges of influence. In other words, if an HFRS outbreak occurs at one location, it is necessary for the public health management to focus on in-situ HFRS prevention and control, as a measure to prevent further spread. This also reflects that the main pathway of HFRS infection is from rodents to humans, rather from person-to-person interaction [6]. In the latter case, an HFRS outbreak would spread in much larger areas. Using the findings of spatiotemporal HFRS spread patterns, public health management can also determine the critical temporal and spatial scales for HFRS prevention and control. For example, if HFRS incidences at some location are identified to belong to class 2, effective measures should be taken for a period of approximately three months and within a radial distance of approximately 50 km (see Fig 7b).

Certain quantitative findings of this work are in line with previous qualitative assessments. For example, based on the finding of the previous studies HFRS incidences are closely associated with environmental factors, a finding that confirms, in part, the suggestions made by [4, 19, 43, 44]. Given BME’s methodological features to assimilate secondary information and auxiliary variables (e.g., [45]), future work should focus on integrating environmental factors into BME analysis, to better understand HFRS spread patterns and provide more accurate predictions. The approach introduced in this work is likely to be valuable in comparisons of spatiotemporal incidence patterns at other regions of China or worldwide. Also, the same methodology could be used in the spatiotemporal modeling and mapping of other epidemics under similar in situ conditions, in which case the choice of the disease incidence classes could be linked to the specific public health purposes.

Future work should also focus on the current shortcomings of the proposed approach. One of them is the need for developing HFRS transition probabilities tests that could lead to stronger inferences regarding the interclass interactions suggested by these probabilities. Another is the relaxation of the space-time homostationarity assumption concerning HFRS spread, which may allow a more detailed assessment of the spatial anisotropy of transition probabilities as described by the HFRS indicators. Lastly, it must be kept in mind that real-world computations usually process datasets of potentially widely varying levels of uncertainty. As a result, in most cases the computational results may not satisfy exactly the theoretical assumptions. Instead, they are expected to strike a balance between theoretical rigor and computational cost is acceptable for practical purposes, which leaves room for potential improvement of the computational component of the proposed analysis.

## Supporting information

S1 Text Space-time points. (DOC)

S2 Text Symbol explanations of data categorization. (DOC)

S3 Text HFRS data pre-processing. (DOC)

S4 Text BME method. (DOC)

S5 Text Characteristics of the stochastic indicator of HFRS incidence. (DOC)

S6 Text The global size of each incidence class. (DOC)

S7 Text Calculation method of the four stochastic indicators. (DOC)

S8 Text Characteristics of JIP values of HFRS in Heilongjiang Province during 2005–2013. (DOC)

S9 Text Computational proof of theory between indicators. (DOC)

S10 Text Space-time values of the four stochastic HFRS indicators. (DOC)

S1 Fig An illustration of the HFRS incidences configuration involving the Tonghe and Boli counties during November 2011 and December 2011. (TIF)

S2 Fig Distribution of HFRS in 2005. (TIF)

S3 Fig Distribution of HFRS in 2006. (TIF)

S4 Fig Distribution of HFRS in 2007. (TIF)

S5 Fig Distribution of HFRS in 2008. (TIF)

S6 Fig Distribution of HFRS in 2009. (TIF)

S7 Fig Distribution of HFRS in 2010. (TIF)

S8 Fig Distribution of HFRS in 2011. (TIF)

S9 Fig Distribution of HFRS in 2012. (TIF)

S10 Fig Distribution of HFRS in 2013. (TIF)

S11 Fig Plots of mean distances across HFRS incidence patches during 2005–2013. (TIF)

S12 FigPlots of $P[\bar{X}(t)\in I_{m}]$ in Heilongjiang Province during 2005–2013 when (a) *X*(*p*) ∈ *I*_1_, (b) *X*(*p*) ∈ *I*_2_, (c) *X*(*p*) ∈ *I*_3_, and (d) *X*(*p*) ∈ *I*_4_.(TIF)

S13 Fig Monthly variation of the JIP sill and range during the period 2005–2013. (TIF)

S14 Fig Plots of the averages of JIP sill and range in the same month (January-December) of each year for the period 2005–2013. (TIF)

S15 Fig The mean interclass (i.e., *m* = *n*) (*a*) JIP, (*b*) IIP, (*c*) IEP and (*d*) SIC plots in Heilongjiang province during 2005–2013 as functions of *h* and *τ*. (TIF)

S16 Fig The mean interclass (*a*) JIP, (*b*) IIP, (*c*) EIP and (*d*) SIC plots (*m* < *n*), and (*e*) JIP, (*f*) IIP, (*g*) EIP and (*h*) SIC plots (*m* > *n*) in Heilongjiang province during 2005–2013 as functions of *h* and *τ*. (TIF)

S17 Fig Plots of the differences EIP-JIP with (*a*) *m* = 1, *n* = 2, (*b*) *m* = 3, *n* = 4, and (c) *m* = 4, *n* = 2 in Heilongjiang province during 2005–2013 as functions of *h* and *τ*. (TIF)

S1 Table Summary statistics of HFRS incidence data in Heilongjiang province before and after log-transformation. (DOCX)

S2 Table Spatial and temporal dependency ranges and fitted theoretical covariance models of the four HFRS incidence classes. (DOCX)

S3 Table Accuracy performance of class-dependent, standard BME and IDW implementations in HFRS incidence estimation. (DOCX)

S4 Table Performance of the three HFRS incidence mapping methods for each class. (DOCX)

S5 Table Space-time averaged JIP values of the four HFRS classes. (DOCX)

S6 Table Space-time averaged IIP values of the four HFRS classes. (DOCX)

S7 Table Space-time averaged EIP values of the four HFRS classes. (DOCX)

S8 Table Space-time averaged SIC values of the four HFRS classes. (DOCX)

S9 Table JIP values of the four HFRS incidence classes (*h* = 0, *τ* = 0). (DOCX)

S10 Table IIP values of the four HFRS incidence classes (*h* = 0, *τ* = 0). (DOCX)

S11 Table EIP values of the four HFRS incidence classes (*h* = 0, *τ* = 0). (DOCX)

S12 Table SIC values of the four HFRS incidence classes (*h* = 0, *τ* = 0). (DOCX)
