# Supplementary material for: Probabilistic logic analysis of the highly heterogeneous spatiotemporal HFRS incidence distribution in Heilongjiang province (China) during 2005-2013
Source: PLoS Negl Trop Dis. 2019 Jan 31;13(1):e0007091. doi: 10.1371/journal.pntd.0007091 (PMC6380603; doi:10.1371/journal.pntd.0007091)
Supplement: S1 Text — (DOC) [file pntd.0007091.s001.doc]

**S1 Text Space-time points**

Let $X(p)$ denote the HFRS incidence at the space-time point $p=(\mathbf{s},t)$, where $\mathbf{s}=(s_{1},s_{2})$ is the location vector and $t$ the time instant. For a visual illustration, in the case of the HFRS distributions displayed in S1 Fig, the $\mathbf{s}_{Tonghe}=(s_{1, Tonghe}, s_{2, Tonghe})$ and $\mathbf{s}_{Boli}=(s_{1, Boli}, s_{2, Boli})$ denote the locations of the Tonghe and Boli counties, respectively, of the Heilongjiang province, and $t_{Nov©11}$ and $t_{Dec©11}$ denote the months of November 2011 and December 2011, respectively.
