# Supplementary material for: Probabilistic logic analysis of the highly heterogeneous spatiotemporal HFRS incidence distribution in Heilongjiang province (China) during 2005-2013
Source: PLoS Negl Trop Dis. 2019 Jan 31;13(1):e0007091. doi: 10.1371/journal.pntd.0007091 (PMC6380603; doi:10.1371/journal.pntd.0007091)
Supplement: S2 Text — (DOC) [file pntd.0007091.s002.doc]

**S2 Text Symbol explanations of data categorization**

The categorical HFRS incidences can be denoted as

$X(p)\in I_{m}$,

which means that the HFRS incidence at point $p$ belongs to the incidence class $I_{m}$, or $X(p')\in I_{n}$, which means that the incidence at point $p'$ belongs to the class $I_{n}$; whereas

$$X(p)\notin I_{m}$$

means that the HFRS incidence at point $p$ does not belong to $I_{m}$, and $X(p')\notin I_{n}$ means that the incidence at point $p'$ does not belong to $I_{n}$ etc. Categorical HFRS incidence variations can be considered within the same class (intraclass incidence variation) and between different classes (interclass incidence variation). For illustration, in the case of S1 Fig, the corresponding categorical incidences belonging to, say, classes $I_{3}$ and $I_{4}$ are denoted as $X(\mathbf{s}_{Tonghe},t_{Nov©11})\in I_{3}$ and $X(\mathbf{s}_{Boli},t_{Dec©11})\in I_{4}$, respectively.
