# Supplementary material for: Probabilistic logic analysis of the highly heterogeneous spatiotemporal HFRS incidence distribution in Heilongjiang province (China) during 2005-2013
Source: PLoS Negl Trop Dis. 2019 Jan 31;13(1):e0007091. doi: 10.1371/journal.pntd.0007091 (PMC6380603; doi:10.1371/journal.pntd.0007091)
Supplement: S3 Text — (DOC) [file pntd.0007091.s003.doc]

**S3 Text HFRS data pre-processing**

The variability of the recorded HFRS incidence data is remarkable (ranging from 0 to 26.79 cases per 10^5^ capita, see S1 Table). Specifically, the highest HFRS incidence value of 26.79 cases per 10^5^ capita, was observed at Youyi county in November 2007; the second and third largest incidence values, 25.90 and 25.65 cases per 10^5^ capita, were recorded at Youyi County in December 2007 and at Baoqing County in November 2005, respectively. On the other hand, the smallest possible incidence value (0 cases per 10^5^ capita) was observed at least at one country of Heilongjiang province during each month. The summary statistics of the original and the log-transformed HFRS incidence data in Heilongjiang province are also shown in S1 Table.

Considering that 58% of the original data are 0s, we divided the rest 42% of the data into four classes, and added the 0-valued data to the first class, i.e., 0-68.5%, 68.5%-79%, 79%-89.5, and 89.5-100%, see Table 1. Evidently, the variability of HFRS incidences in each class is much smaller than the variability in the original dataset (S1 Table). The HFRS incidences in class $I_{4}$ exhibit the largest variation compared to the remaining three classes.

As was noticed in the main text, the implementation of the three class selection criteria suggested that the optimal choice was to use four HFRS classes (categories). Indeed, numerical tests were performed that considered the possibility of selecting fewer or more classes. As it turned out:

If three HFRS classes are selected, the resulting HFRS maps are inferior in terms of accuracy (MAE, RMSE and R^2^ values) compared to the maps generated if four HFRS classes are selected, instead. Moreover, if we divide the original HFRS data into three classes, there will be 1372 space-time points out of the total number of original data (14040 space-time points) that cannot be estimated at the cross validation stage, whereas if 4 classes are considered, only 22 space-time points out of the 14040 cannot be estimated in the cross validation stage.

If five or more classes were selected this choice will violate the class selection criteria. In particular: Criterion *ii* is violated, i.e., one class will only have 72 data locations, which is less than the required 60% cover of the area=78 locations. Criterion *iii* is also violated, i.e., there are several mapping grid nodes that are surrounded by an insufficient or even zero number of data, thus making incidence estimation and mapping impossible. Yet another problem is that if we divide the original data into five classes, there will be 2228 space-time points out of the total number of original data (14040 space-time points) that cannot be estimated at the cross validation stage (we recall that if 4 classes are considered, only 22 space-time points out of the 14040 that cannot be estimated in the cross validation stage).

Hence, it was concluded that the optimal number of HFRS classes determined by the three criteria discussed earlier is four
