# Supplementary material for: Probabilistic logic analysis of the highly heterogeneous spatiotemporal HFRS incidence distribution in Heilongjiang province (China) during 2005-2013
Source: PLoS Negl Trop Dis. 2019 Jan 31;13(1):e0007091. doi: 10.1371/journal.pntd.0007091 (PMC6380603; doi:10.1371/journal.pntd.0007091)
Supplement: S4 Text — (DOC) [file pntd.0007091.s004.doc]

**S4 Text BME method**

Here we only present a brief outline of the BME method. Regarding the general knowledge base (G-KB), the theoretical mean and covariance models for log-transformed HFRS incidence $Y(p)$, namely

$\begin{matrix} m_{Y}(p)=\overline{Y(p)}, \\ c_{Y}(|h|,\tau)=c_{Y}(p,p')=\overline{[Y(p)-m_{Y}(p)][Y(p')-m_{Y}(p')]}, \end{matrix}$ (S1a-b)

were introduced to describe the spatiotemporal structure of HFRS incidences, where $h=\mathbf{s}'-\mathbf{s}$ and $\tau=t'-t$ denote the vector (spatial) distance and the time separation, respectively, between the space-time points $p$ and $p'$, and $|h|$ is the magnitude of the vector distance. Generally, the shape of the covariance function $c_{Y}(|h|,\tau)$ provides information on the magnitude of dependence of HFRS incidences, as a function of $|h|$ and $\tau$. To what concerns site-specific information, the recorded HFRS incidences in each county were integrated in the BME framework as site-specific knowledge base (S-KB). Thus, HFRS incidence estimation in the BME context is conducted by solving the system of equations

$\begin{matrix} \int d\chi(g-\bar{\mathbf{g}})e^{\mu\cdot g}=0, \\ \int d\chi\xi e^{\mu\cdot g}-af_{K}=0, \end{matrix}$ (S2a-b)

where the vector function ***g*** expresses the available general knowledge bases, $\bar{\mathbf{g}}$ represents the mean value of ***g***, the function $\xi$ expresses the available site-specific knowledge base; $\mu$ is a vector of coefficients representing the relative importance of different elements in ***g***; $\chi$ represents realizations of HFRS incidences, *a* is a normalization parameter, and $f_{k}$ is the probability density function of HFRS incidences at unsampled locations used to obtain $\chi_{k}$.
