# Supplementary material for: Probabilistic logic analysis of the highly heterogeneous spatiotemporal HFRS incidence distribution in Heilongjiang province (China) during 2005-2013
Source: PLoS Negl Trop Dis. 2019 Jan 31;13(1):e0007091. doi: 10.1371/journal.pntd.0007091 (PMC6380603; doi:10.1371/journal.pntd.0007091)
Supplement: S5 Text — (DOC) [file pntd.0007091.s005.doc]

**S5 Text Characteristics of the stochastic indicator of HFRS incidence**

*1. The joint incidence probability*

If space-time homostationarity is assumed (i.e., the HFRS incidence distribution is space homogeneous and time stationary), the indicators are functions of $\Delta\mathbf{p}=\mathbf{p}-\mathbf{p}'=(\mathbf{s}-\mathbf{s}',t-t')=(\mathbf{h},\tau)$. For example, the JIP can be written as

$JIP_{X}^{m,n}(\mathbf{p}, \mathbf{p}')=JIP_{X}^{m,n}(\Delta\mathbf{p})=JIP_{X}^{m,n}(\mathbf{h},\tau)$ (S3)

for all $p$ and $p'$ such that $\Delta\mathbf{p}=(\mathbf{h},\tau)$. Isotropy further implies that the JIP is only a function of the length $|\mathbf{h}|$ and time separation $\tau$, i.e., the JIP is a function of $|\Delta\mathbf{p}| =(|\mathbf{h}|,\tau)$. The interpretation of Eq (S3) is that, in probability terms, $P[X(\mathbf{p})\in I_{m}\wedge X(\mathbf{p}')\in I_{n}]=P[X(\mathbf{p}')\in I_{n}\wedge X(\mathbf{p})\in I_{m}]$ for all $p$ and $p'$ such that $\Delta\mathbf{p}=(\mathbf{h},\tau)$. For illustration (S1 Fig), the probability that both categorical incidences $X(\mathbf{s}_{Tonghe},t_{Nov©11})\in I_{3}$ and $X(\mathbf{s}_{Boli},t_{Dec©11})\in I_{4}$ occur is a function of the distance ${}^{3,4}\mathbf{h}_{Tonghe-Boli}$ between the Tonghe and Boli counties and the time separation ${}^{3,4}\tau_{Nov-Dec©11}$ between December and November 2011. Homostationarity also implies that for all $p$, it is valid that

$JIP_{X}^{m,m}(0,0)=P[X(p)\in I_{m}]=℘_{m}$,

where is a class ($I_{m}$)-dependent but space- and time-independent probability. This assumption becomes stricter if we replace $\Delta\mathbf{p}=(\mathbf{h},\tau)$ with $|\Delta\mathbf{p}| =(|\mathbf{h}|,\tau)$, which is referred to as the isostationary assumption. In practice, the homostationarity assumption is satisfied after a certain transformation (e.g., detrending) of the original dataset.

*2. The incidence implication probability*

Like in the JIP case, if space-time homostationarity is assumed, then the IIP can be written as

$IIP_{X}^{m,n}(\mathbf{p}, \mathbf{p}')=IIP_{X}^{m,n}(\mathbf{h},\tau)$, (S4)

for all $p$ and $p'$ such that $\Delta\mathbf{p}=(\mathbf{h},\tau)$. In the case of isotropy, the IIP is a function of $|\Delta\mathbf{p}| =(|\mathbf{h}|,\tau)$. For illustration, this means that the probability $X(p)\in I_{m}\to X(p')\in I_{n}$ is a function of the distance between the Tonghe and Boli counties and the time separation between December and November 2011 (S1 Fig). Since IIP is related to JIP by $IIP_{X}({}^{m}\mathbf{p},{}^{n}\mathbf{p}')$ $=JIP_{X}({}^{m}\mathbf{p},{}^{n}\mathbf{p}')+P[X(p)\notin I_{m}]$, the JIP homostationarity (isostationarity) implies that of IIP; in this case,

$IIP_{X}^{m,n}(\mathbf{h},\tau)=JIP_{X}^{m,n}(\mathbf{h},\tau)+1-℘_{m}$, (S5)

i.e., the IIP of incidence spread is larger than the corresponding JIP by an amount equal to the probability that $X(p)\notin I_{m}$, which plays a pivotal role in the determination of the implication probability of HFRS spread (i.e., the IIP provides a more broad assessment of the JIP probability, since the former includes incidence possibilities that are not included in the latter). This means, e.g., that if the incidence $X(\mathbf{s}_{Tonghe},t_{Nov©11})\in I_{3}$ is highly unlikely, i.e., $P[X(\mathbf{s}_{Tonghe},t_{Nov©11})\notin I_{3}]=1-℘_{3}$ is very high, then the $IIP_{X}({}^{3,4}\mathbf{h},{}^{3,4}\tau)$ can be very large. Consequently, from a very unlikely HFRS incidence $X(\mathbf{s}_{Tonghe},t_{Nov©11})\in I_{3}$, one can draw a conclusion about the incidence $X(\mathbf{s}_{Boli},t_{Dec©11})\in I_{4}$ with a high degree of probability.

*3. The equivalency* *incidence probability*

If space-time homostationarity and isotropy is assumed for the HFRS distribution, then the EIP can be written accordingly as

$EIP_{X}^{m,n}(\mathbf{p}, \mathbf{p}')=EIP_{X}^{m,n}(\mathbf{h},\tau)$, (S6)

for all $p$ and $p'$ such that $\Delta\mathbf{p}=(\mathbf{h},\tau)$. Since EIP is related to JIP by

$EIP_{X}^{m,n}(\mathbf{h},\tau)=2JIP_{X}^{m,n}(\mathbf{h},\tau)+1-℘_{m}-℘_{n}$, (S7)

the JIP homostationarity (isostationarity) implies that of EIP, and the theoretical Eq (S7) holds in all Heilongjiang cases considered in result Fig 6. This means, e.g., that if the incidences $X(\mathbf{s}_{Tonghe},t_{Nov©11})\in I_{3}$ and $X(\mathbf{s}_{Boli},t_{Dec©11})\in I_{4}$ are highly unlikely, i.e., $P[X(\mathbf{s}_{Tonghe},t_{Nov©11})\in I_{3}]=℘_{3}$ and $P[X(\mathbf{s}_{Boli},t_{Dec©11})\in I_{4}]=℘_{4}$ are very small, then the $EIP_{X}({}^{3,4}\mathbf{h},{}^{3,4}\tau)$ can be very large. Hence, and play a significant role in the determination of the equivalent probability of HFRS spread.

In general, the JIP, IIP and EIP indicators of different configurations of space-time points and incidence classes may differ from each other, i.e., the specific configurations of the locations $\mathbf{s}$ and $\mathbf{s}'$, the time instants $t$ and $t'$, or the classes $I_{m}$ and $I_{n}$ may affect the interpretation of the stochastic indicators. For illustration, in the case of the HFRS distributions displayed in S1 Fig let $\mathbf{s}_{Tonghe}$ and $\mathbf{s}_{Boli}$ denote the spatial locations of the Tonghe and Boli counties, respectively, $t_{Nov©11}$ and $t_{Dec©11}$ denote the time instants of November 2011 and December 2011, respectively. Then, the JIP that $X(\mathbf{s}_{Tonghe},t_{Nov©11})\in I_{3}$ (i.e., the HFRS incidence in the Tonghe county during November 2011 belongs to class $I_{3}$) and $X(\mathbf{s}_{Boli},t_{Dec©11})\in I_{4}$ (the Boli county incidence during December 2011 belongs to class $I_{4}$) is different from the JIP that $X(\mathbf{s}_{Tonghe},t_{Nov©11})\in I_{4}$ (the Tonghe county incidence during November 2011 belongs to class $I_{4}$) and $X(\mathbf{s}_{Boli},t_{Dec©11})\in I_{3}$ (the Boli county incidence during December 2011 belongs to class $I_{3}$). In numerical terms, it was calculated that the corresponding joint probability

$$JIP_{X}^{3,4}(\mathbf{h}_{Boli-Tonghe}\wedge\tau_{Dec©11-Nov©11})=P[X(\mathbf{s}_{Tonghe},t_{Nov©11})\in I_{3}, X(\mathbf{s}_{Boli}\wedge t_{Dec©11})\in I_{4}]=0.0019$$

is different than the joint probability

$JIP_{X}^{4,3}(\mathbf{h}_{Boli-Tonghe}\wedge\tau_{Dec©11-Nov©11})=P[X(\mathbf{s}_{Tonghe},t_{Nov©11})\in I_{4}\wedge X(\mathbf{s}_{Boli},t_{Dec©11})\in I_{3}]=0.0020$,

where $\mathbf{h}_{Boli-Tonghe}=\mathbf{s}_{Boli}-\mathbf{s}_{Tonghe}$ and $\tau_{Dec©11-Nov©11}=t_{Dec©11}-t_{Nov©11}$.

Similarly, the HFRS indicators among different configurations of space-time points and incidence classes may differ from each other, yet they are linked to each other. For example, the IIP of the categorical HFRS incidence $X(\mathbf{s}_{Tonghe},t_{Nov©11})\in I_{3}$ implying logically the incidence $X(\mathbf{s}_{Boli},t_{Dec©11})\in I_{4}$ is equal to the IIP of the categorical incidence $X(\mathbf{s}_{Boli},t_{Dec©11})\in I_{3}$ implying the categorical incidence $X(\mathbf{s}_{Tonghe},t_{Nov©11})\in I_{4}$ plus the difference between the probability that $X(\mathbf{s}_{Boli},t_{Dec©11})\in I_{4}$ and the probability that $X(\mathbf{s}_{Tonghe},t_{Nov©11})\in I_{3}$. Expressed in formal terms,

$$IIP_{X}^{3,4}(\mathbf{h}_{Boli-Tonghe},\tau_{Dec©11-Nov©11})=IIP_{X}^{4,3}(\mathbf{h}_{Boli-Tonghe},\tau_{Dec©11-Nov©11})+P[X(\mathbf{s}_{Boli},t_{Dec©11})\in I_{4}]-$$

$P[X(\mathbf{s}_{Tonghe},t_{Nov©11})\in I_{3}]=IIP_{X}^{4,3}(\mathbf{h}_{Boli-Tonghe},\tau_{Dec©11-Nov©11})+℘_{4}-℘_{3}$. Therefore, if , then $IIP_{X}^{3,4}(\mathbf{h}_{Boli-Tonghe},\tau_{Dec©11-Nov©11})\geq IIP_{X}^{4,3}(\mathbf{h}_{Boli-Tonghe},\tau_{Dec©11-Nov©11})$, and vice versa. In general, it holds that

$IIP_{X}^{m,n}(p, p')=IIP_{X}^{n,m}(p', p)+P[X(p')\in I_{n}]-P[X(p)\in I_{m}]$ (S8)

for all $p$ and $p'$ in the Heilongjiang province.

*4. Links with statistical incidence conditional*

If space-time homostationarity and isotropy is assumed, then the IEP can be written as

$SIC_{X}^{m,n}(\mathbf{p}, \mathbf{p}')=SIC_{X}^{m,n}(\mathbf{h},\tau)$, (S9)

for all $p$ and $p'$ such that $\Delta\mathbf{p}=(\mathbf{h},\tau)$. Notice that the classes $I_{m}$ and $I_{n}$ are usually assumed mutually exclusive, in which case the conditions $SIC_{X}^{m,m}(p,p)=SIC_{X}^{m,m}(0,0)=1$ and $SIC_{X}^{m,n}(p, p)=SIC_{X}^{m,n}(0,0)=0$ apply, meaning that the corresponding plots of $SIC_{X}^{m,m}(h,\tau)$ and $SIC_{X}^{m,n}(h,\tau)$ as functions of $\mathbf{h}$ and $\tau$ decrease starting at 1 and increase starting at 0, respectively.
