# Supplementary material for: Probabilistic logic analysis of the highly heterogeneous spatiotemporal HFRS incidence distribution in Heilongjiang province (China) during 2005-2013
Source: PLoS Negl Trop Dis. 2019 Jan 31;13(1):e0007091. doi: 10.1371/journal.pntd.0007091 (PMC6380603; doi:10.1371/journal.pntd.0007091)
Supplement: S6 Text — (DOC) [file pntd.0007091.s006.doc]

**S6 Text The global size of each incidence class**

Real-world computations usually process datasets of considerable heterogeneity and with widely varying levels of uncertainty. As a result, in several cases the computational results may not satisfy exactly the theoretical formulas, instead, they are expected to do so at a degree that is sufficient for practical purposes. This situation is particularly valid when the actual mean incidence values over the space-time domain of interest are approximated by numerical averages. Indeed, the various HFRS probabilities can be determined by superimposing a lattice on an incidence map, like that of Fig 6, and calculating the frequency of the HFRS-related event of interest across the lattice cells. For illustration, at a fixed time $t$ the probability of the event “$X(\mathbf{s},t)\in I_{m}$” is the ratio of the area $|A_{t}^{m}|$ where this event occurs over the total area $|A|$, i.e., $P[X(\mathbf{s},t)\in I_{m}]\approx\frac{|A_{t}^{m}|}{|A|}$. Here we notice that, by definition, $P[X(\mathbf{s},t)\in I_{m}]=JIP_{X}^{m}(\mathbf{s},t)$, and that this probability is defined as the probability of occurrence of the event “$X(\mathbf{s},t)\in I_{m}$” regardless of the event’s occurrence in neighboring lattice cells. Several other probabilities can be computed in a similar manner. So, at time *t* let $\bar{X}(t)$ denote the spatial mean of the HFRS incidence $X(\mathbf{s},t)$ averaged over the Heilongjiang region. For each time *t* (month), the probability $P[\bar{X}(t)\in I_{m}]$ represents the geographical fraction of the Heilongjiang region with $\bar{X}(t)\in I_{m}$ ($m=1,2,3,4$), which in practice can be calculated as

$P[\bar{X}(t)\in I_{m}]\approx\frac{1}{|A|}\sum_{\mathbf{s}\in A_{t}^{m}} JIP_{X}^{m}(\mathbf{s},t)$, (S10)

where $|A| =473,000 km^{2}$ is the total Heilongjiang area. S12a-d Figs present the temporal variation of $P[\bar{X}(t)\in I_{m}]$ for each of the four HFRS classes. The $\overline{JIP}_{X}^{m}(t)$ values are the average values of the fractions $P[\bar{X}(t)\in I_{m}]$ for the entire period 2005-2013, i.e., $℘_{m}=\overline{P[\bar{X}(t)\in I_{m}]}$, where the second bar here denotes the temporal averaging of $P[\bar{X}(t)\in I_{m}]$ over the 108 months of the period 2005-2013, so that

(S11)

can be considered constant (space- and time-independent) for the classes $I_{m}$ ($m=1,2,3,4$).
