# Supplementary material for: Probabilistic logic analysis of the highly heterogeneous spatiotemporal HFRS incidence distribution in Heilongjiang province (China) during 2005-2013
Source: PLoS Negl Trop Dis. 2019 Jan 31;13(1):e0007091. doi: 10.1371/journal.pntd.0007091 (PMC6380603; doi:10.1371/journal.pntd.0007091)
Supplement: S7 Text — (DOC) [file pntd.0007091.s007.doc]

**S7 Text Calculation method of the four stochastic indicators**

For convenience let $SII_{X}=JIP_{X}, IIP_{X}, EIP_{X}, SIC_{X}$ denote the four stochastic incidence indicators (SII) considered in this work. In theory, these $SII_{X}$ provide different yet complementary perspectives concerning the probability of transition from one incidence class to another. In practice, as above, the various probabilities signified by the $SII_{X}$ can be determined by superimposing a lattice on an incidence map. Then, for each $SII_{X}$ the HFRS transition frequencies between the incidence classes along a specified direction or omnidirectionally are calculated by counting the times a given class is followed by itself or by another class on the lattice and then dividing by the total number of transitions $N_{\mathbf{h}, \tau}$, i.e.,

. (S12)

The different perspectives signified by $SII_{X}$ and their numerical plots in the case of the Heilongjiang study of the present work are studied next.
