# Supplementary material for: Probabilistic logic analysis of the highly heterogeneous spatiotemporal HFRS incidence distribution in Heilongjiang province (China) during 2005-2013
Source: PLoS Negl Trop Dis. 2019 Jan 31;13(1):e0007091. doi: 10.1371/journal.pntd.0007091 (PMC6380603; doi:10.1371/journal.pntd.0007091)
Supplement: S8 Text — (DOC) [file pntd.0007091.s008.doc]

**S8 Text Characteristics of JIP values of HFRS in Heilongjiang Province during 2005-2013**

Interestingly, by comparing the JIP surfaces, we found that $JIP_{X}^{m,n}(p, p')=JIP_{X}^{n,m}(p, p')$ only if $\tau=t-t'=0$, i.e., if the same month is considered by these two HFRS dependence surfaces. In this case, we can say that the categorical HFRS incidences at space-time points $\mathbf{p}$ and $\mathbf{p}'$ and the corresponding incidence classes $I_{m}$ and $I_{n}$are exchangeable (i.e., we can write that $P[X(\mathbf{s},t)\in I_{m}, X(\mathbf{s}+\mathbf{h},t+\tau)\in I_{n}]=$ $P[X(\mathbf{s}+\mathbf{h},t+\tau)\in I_{m},X(\mathbf{s},t)\in I_{n}]$). In the other cases, the joint probability difference $|JIP_{X}^{m,n}(p,p')-JIP_{X}^{n,m}(p,p')|$ increases with increasing time separation $t-t'$, i.e., the longer the separation time between two HFRS incidences is, the larger is the difference between the joint probabilities of any pair of incidence classes considered.

Selecting the upper limit of class 1 (i.e. the HFRS incidence value 0.3576) as the threshold value, the JIP values, range and sill were calculated based on the 108 maps generated by BME, see plots in S13 Fig. In this case, the JIP value is the probability that at both $p$ and $p'$ points the HFRS incidences were both larger than 0.3576. We observe that the plots of the JIP sill and range fluctuate around a constant mean sill and a constant range, respectively. We also observe that during the June and November of each year considered, the JIP sill and range reach their peaks, which is consist with the corresponding plots of the covariance sill and range (Fig 3) of covariance calculated by the original incidence data without transformation. Also, S14 Fig presents the JIP sill and range averages of the same month (January-December) during the period 2005-2013. The shapes of these plots suggest that the values of the JIP sill and range fluctuate during the same time period and that these fluctuations follow similar patterns, overall.
