# Supplementary material for: Probabilistic logic analysis of the highly heterogeneous spatiotemporal HFRS incidence distribution in Heilongjiang province (China) during 2005-2013
Source: PLoS Negl Trop Dis. 2019 Jan 31;13(1):e0007091. doi: 10.1371/journal.pntd.0007091 (PMC6380603; doi:10.1371/journal.pntd.0007091)
Supplement: S9 Text — (DOC) [file pntd.0007091.s009.doc]

**S9 Text Computational proof of theory between indicators**

S15a-c Figs present plots of the mean intraclass surfaces (i.e., $m=n$) of the four indicators as functions of the space-time lags $\mathbf{h}, \tau$, which were calculated as the corresponding averages of $JIP_{X}^{m,m}(\mathbf{h},\tau)$, $IIP_{X}^{m,m}(\mathbf{h},\tau)$, $EIP_{X}^{m,m}(\mathbf{h},\tau)$ and $SIC_{X}^{m,m}(\mathbf{h},\tau)$, with $m=1, 2, 3, 4$. The shapes of the four surfaces look similar, however, the corresponding numerical indicator values differ. Overall, it is valid that

$JIP_{X}<EIP_{X}<IIP_{X}$, and $JIP_{X}<SIC_{X}$.

Interpretationally, these inequalities imply that if pair of space-time points $p$ and $p+\mathbf{h}$ are randomly selected in the study domain the probability that both categorical incidences $X(p)\in I_{m}$ and $X(p+\mathbf{h})\in I_{m}$ occur is smaller than the probability that the incidences $X(p)\in I_{m}$ and $X(p+\mathbf{h})\notin I_{m}$ do not occur, which, in turn is smaller than the probability that $X(p)\in I_{m}$ and $X(p+\mathbf{h})\in I_{m}$ either both occur or both do not occur; and, the probability that both $X(p)\in I_{m}$ and $X(p+\mathbf{h})\in I_{m}$ occur is smaller than the conditional probability of $X(p+\mathbf{h})\in I_{m}$ given $X(p)\in I_{m}$.

Similarly, S16a-h Figs present plots of the mean interclass JIP, IIP, EIP and SIC surfaces (i.e., $m\neq n$) calculated as the average of the four classes $JIP_{X}^{m,n}(\mathbf{h},\tau)$, $IIP_{X}^{m,n}(\mathbf{h},\tau)$, $EIP_{X}^{m,n}(\mathbf{h},\tau)$ and $SIC_{X}^{m,n}(\mathbf{h},\tau)$, with $m, n=1, 2, 3, 4$, as a function of the space-time lags $\mathbf{h}$ and $\tau$. As should be expected, the JEP and EIP plots are the same for $m<n$ and $m>n$. As regards the IIP and SIC indicators, although their plots look very similar for $m<n$ vs. $m>n$, there are some numerical differences. All mean HFRS indicator plots initially increase as functions of $\mathbf{h}$ and $\tau$, and then they approach some stable (asymptotic) value. The plots of the four indicators offer complementary visualizations of the variation of the different probabilities of transition between incidence classes, i.e., the probabilities with which the different levels of HFRS incidences occur next to each other and so they describe the dependency pattern of the space-time arrangement of the HFRS patches occupied by the different incidence classes.
