# Supplementary material for: Probabilistic logic analysis of the highly heterogeneous spatiotemporal HFRS incidence distribution in Heilongjiang province (China) during 2005-2013
Source: PLoS Negl Trop Dis. 2019 Jan 31;13(1):e0007091. doi: 10.1371/journal.pntd.0007091 (PMC6380603; doi:10.1371/journal.pntd.0007091)
Supplement: S10 Text — (DOC) [file pntd.0007091.s010.doc]

**S10 Text Space-time values of the four stochastic HFRS indicators**

The space-time averaged values of the four stochastic HFRS indicators $SII_{X}$ ($=JIP_{X}, IIP_{X}, EIP_{X}, SIC_{X}$) are displayed in S5-8 Tables. These mean values were calculated according to Eq (S12) as

, (S13)

where the bar denotes mean value. In S5 and S7 Tables the interclass JIP and IEP values are relatively symmetric, whereas an interesting feature of S6 and S8 Table is the complete lack of symmetry of the interclass IIP and SIC values, respectively. The elements of S5 Table sum up to 1, whereas the elements of each row of S8 Table sum up to 1. As was mentioned earlier, each one of the four stochastic HFRS indicators provides a different perspective concerning the probability of transition from one incidence class to another (i.e., we distinguished between joint, implication equivalence and conditional probabilities). For example, in S6 Table we see that the implication probabilities of transition from every incidence class to class $I_{1}$ are large (some close to 1) signifying that, in the IIP sense of incidence transition, the classes ($I_{2}$,$I_{3}$ and $I_{4}$) occur somehow as “islands” surrounded by incidence patches of class $I_{1}$. On the other hand none of the implication probabilities from incidence class $I_{1}$ to other classes do.

The different intraclass and interclass transition probabilities of the four indicators in S5-8 Tables convey valuable complementary information of the HFRS spread in the Heilongjiang province during 2005-2013. The study of these tables provides can improve our understanding of the HFRS spread in the domain of interest. For illustration, let us consider a few numerical examples. If a pair of space-time points $p$ and $p'$ are selected randomly in Heilongjiang province during 2005-2013, then certain useful probabilities are readily available from S5-8 Tables concerning the interclass HFRS incidences $X(p)\in I_{2}$ and $X(p')\in I_{1}$ at these two points In particular, the probability that:

- “both interclass HFRS incidences occur” is very low (0.132, in S5 Table);

- “either the HFRS occurs at $p'$ or it does not that at $p$” is very high (0.878, in S6 Table);

- “HFRS at $p'$ occurs given that it occurs at $p$” is relatively high (0.517, in S8 Table);

- “either both HFRS incidences occur or both do not” is moderate (0.368, in S7 Table).

These four different probabilities offer complementary quantitative assessments of the incidence dependency between any pair of points in the space-time domain of interest, with relative significance depending on the HFRS perspective adapted. Also, as can be seen in S5-S8 Tables, some order relations can be established between the four categorical HFRS indicators above. For example, given the classes $I_{m}$, $I_{n}$ and $I_{q}$ ($m,n,q=1,2,3,4$), then $SIC_{X}^{m,n}(p_{1}, p_{2})>SIC_{X}^{m,q}(p_{1}, p_{3})$ if and only if $IIP_{X}^{m,n}(p_{1}, p_{2})>IIP_{X}^{m,q}(p_{1}, p_{3})$.

Since the stochastic HFRS indicators act complementary, it may be instructive to compare them numerically. For illustration, in S17 Fig we plot the differences $EIP_{X}({}^{m,n}\mathbf{h},{}^{m,n}\tau)-JIP_{X}({}^{m,n}\mathbf{h},{}^{m,n}\tau)$ as functions of $\mathbf{h}$ and $\tau$ for different combinations of $m$ and $n$. The EIP-JIP differences are always positive, as was expected. But the shapes of the EIP-JIP plots differ considerably depending on the interclass combination of $m$ and $n$. In particular, for $m<n$ the EIP-JIP plot increases with $\mathbf{h}$ and $\tau$ (S17a Fig); whereas for $m>n$ the EIP-JIP plots decrease with $\mathbf{h}$ and slightly increase with$\tau$ (S17b-c Figs).

The numerical results in the present Heilongjiang study satisfy all the theoretical conditions of the HFRS indicators. For illustration, in theory the following conditions are valid concerning the stochastic HFRS indicators:

$IIP_{X}^{m,m}(0,0)=EIP_{X}^{m,m}(0,0)=SIC_{X}^{m,m}(0,0)=1$,

$JIP_{X}^{m,m}(0,0)=℘_{m}$,

$JIP_{X}^{m,n}(0,0)=$ $SIC_{X}^{m,n}(0,0)=0$,

$IIP_{X}^{m,n}(0, 0)=1-℘_{m}$, and

$EIP_{X}^{m,n}(0, 0)=1-(℘_{m}+℘_{n})$.

Indeed, our numerical computations show that the Heilongjiang data satisfy these conditions (see, S9-12 Tables). For example, $IIP_{X}^{1,2}(0, 0)=1-℘_{1}=1-0.6767=0.3233$, $IIP_{X}^{2,1}(0, 0)=1-℘_{1}=$ $1-0.2314=0.7686$, and $EIP_{X}^{1,2}(0, 0)=1-(℘_{1}+℘_{2})=1-0.6767-0.2314=0.0919$. Some more observations can be made concerning the probability values in these tables. With the exception of S10 Table (IIP), the three other tables are symmetric. In S12 Table (SIC), all off-diagonal probabilities are 0, whilst the diagonal probabilities are equal to the corresponding ($m=1,2,3,4$). In S10 Table (IIP), all diagonal probabilities are 1, whilst the off-diagonal probabilities are equal to the corresponding $1-℘_{m}$ ($m=1,2,3,4$). All diagonal probabilities of S11 Table (EIP) are equal to 1, whilst the off-diagonal probabilities are equal to the corresponding $1-℘_{m}-℘_{n}$ ($m,n=1,2,3,4$). And, all the diagonal probabilities of S12 Table (SIC) are equal to 1, but all its off-diagonal probabilities are equal to 0.
