# Supplementary figures and images for: Probabilistic logic analysis of the highly heterogeneous spatiotemporal HFRS incidence distribution in Heilongjiang province (China) during 2005-2013
Source: PLoS Negl Trop Dis. 2019 Jan 31;13(1):e0007091. doi: 10.1371/journal.pntd.0007091 (PMC6380603; doi:10.1371/journal.pntd.0007091)

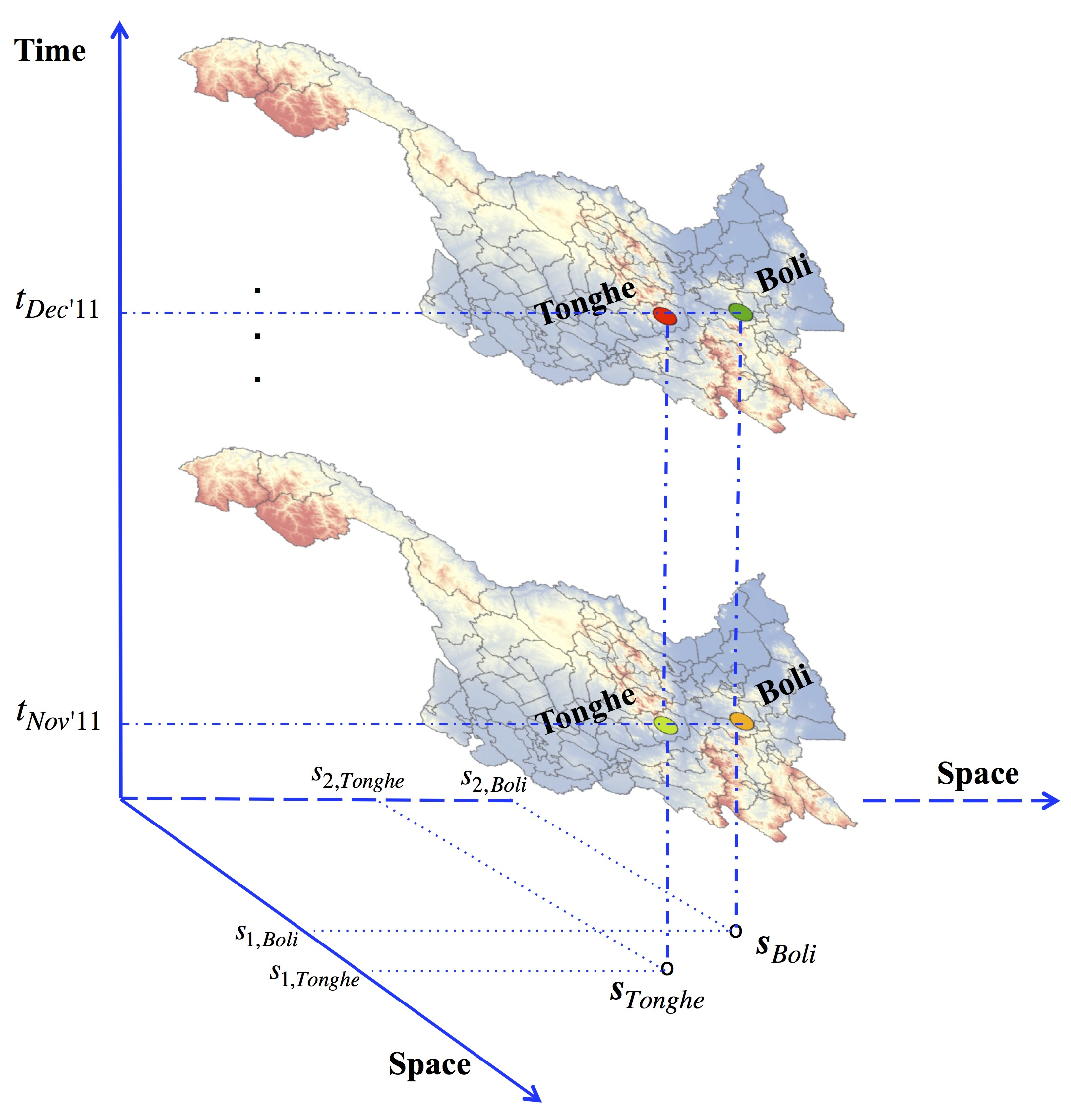

Supplement: S1 Fig — (TIF) [file pntd.0007091.s011.tif]

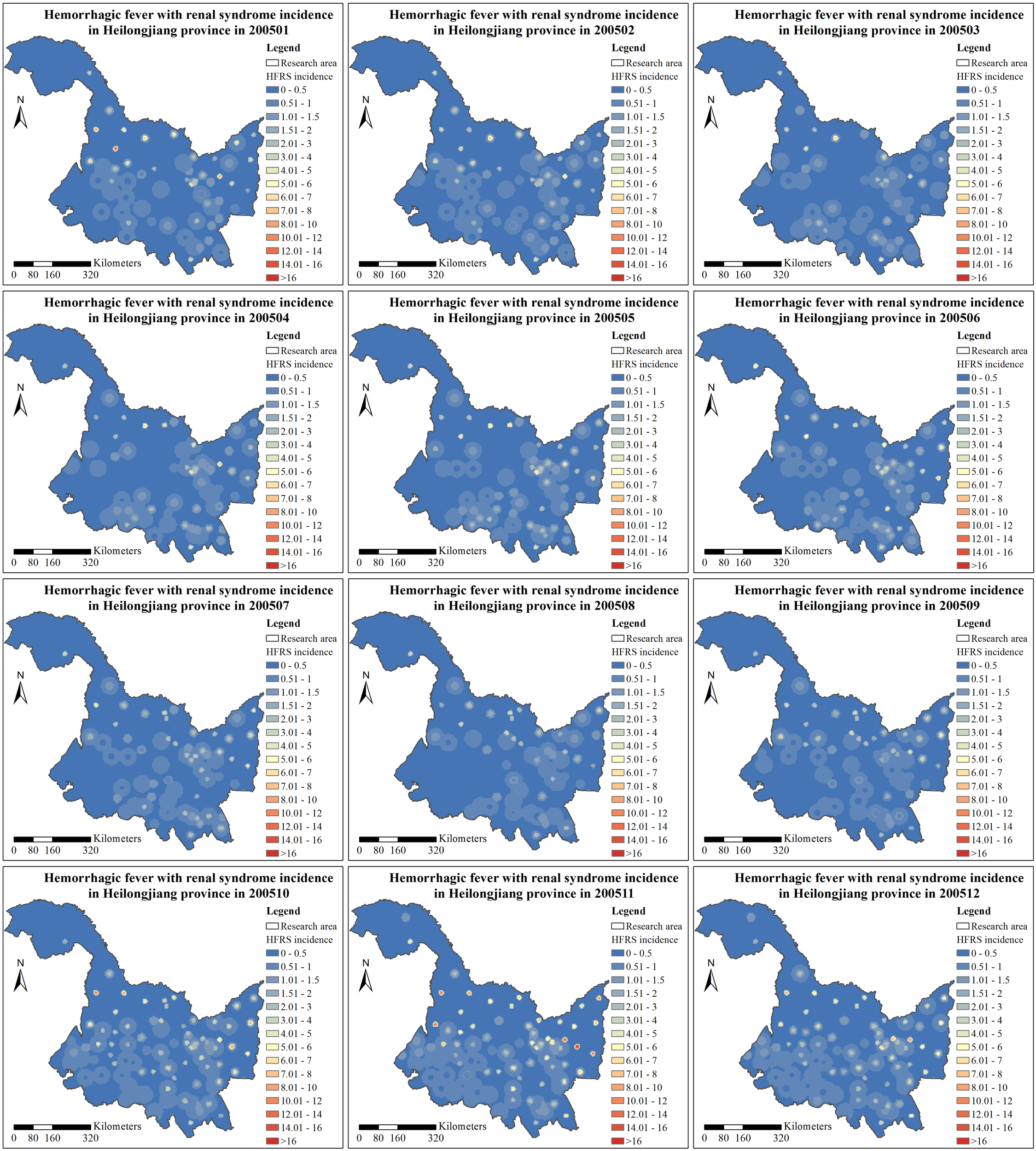

Supplement: S2 Fig — (TIF) [file pntd.0007091.s012.tif]

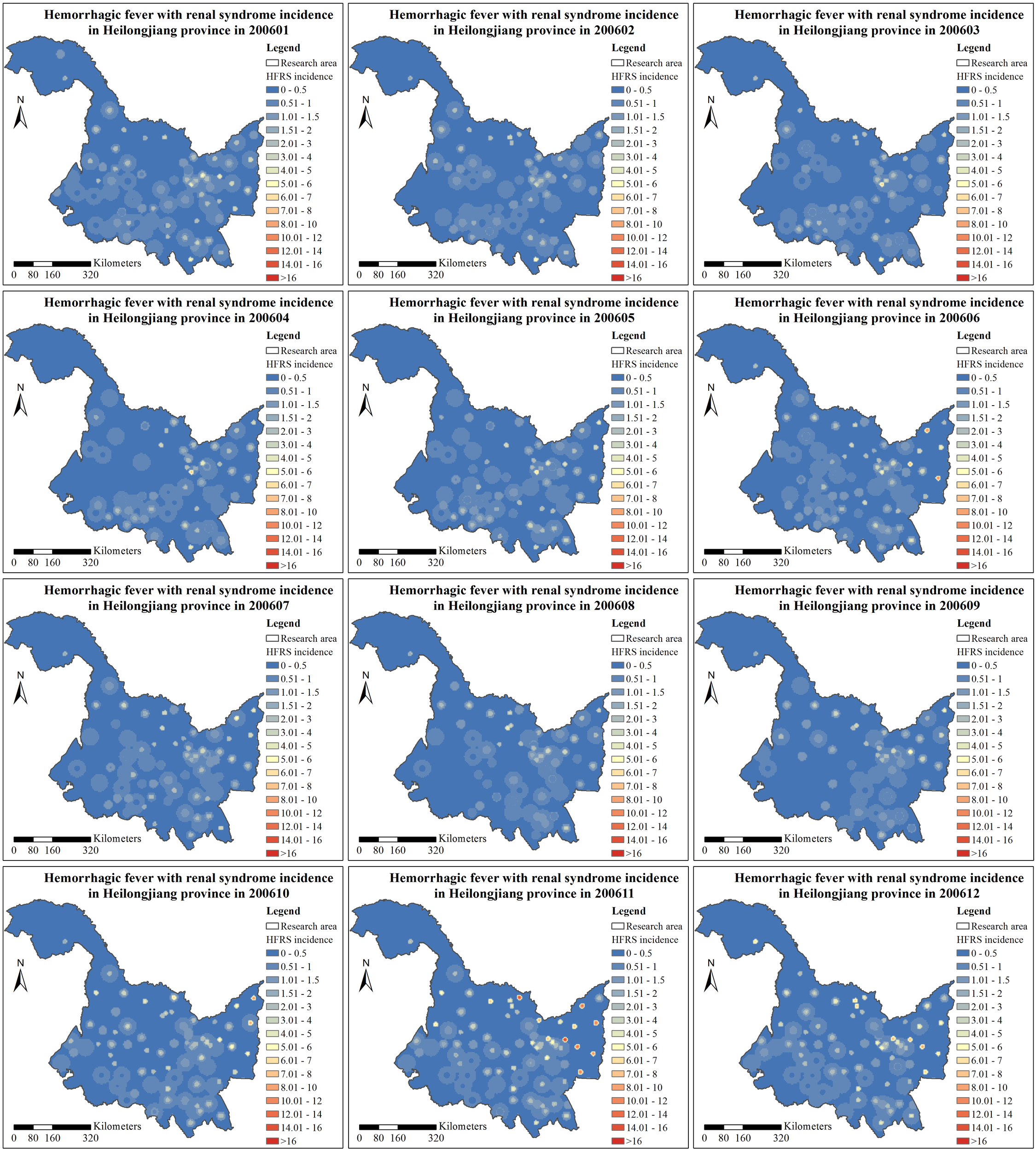

Supplement: S3 Fig — (TIF) [file pntd.0007091.s013.tif]

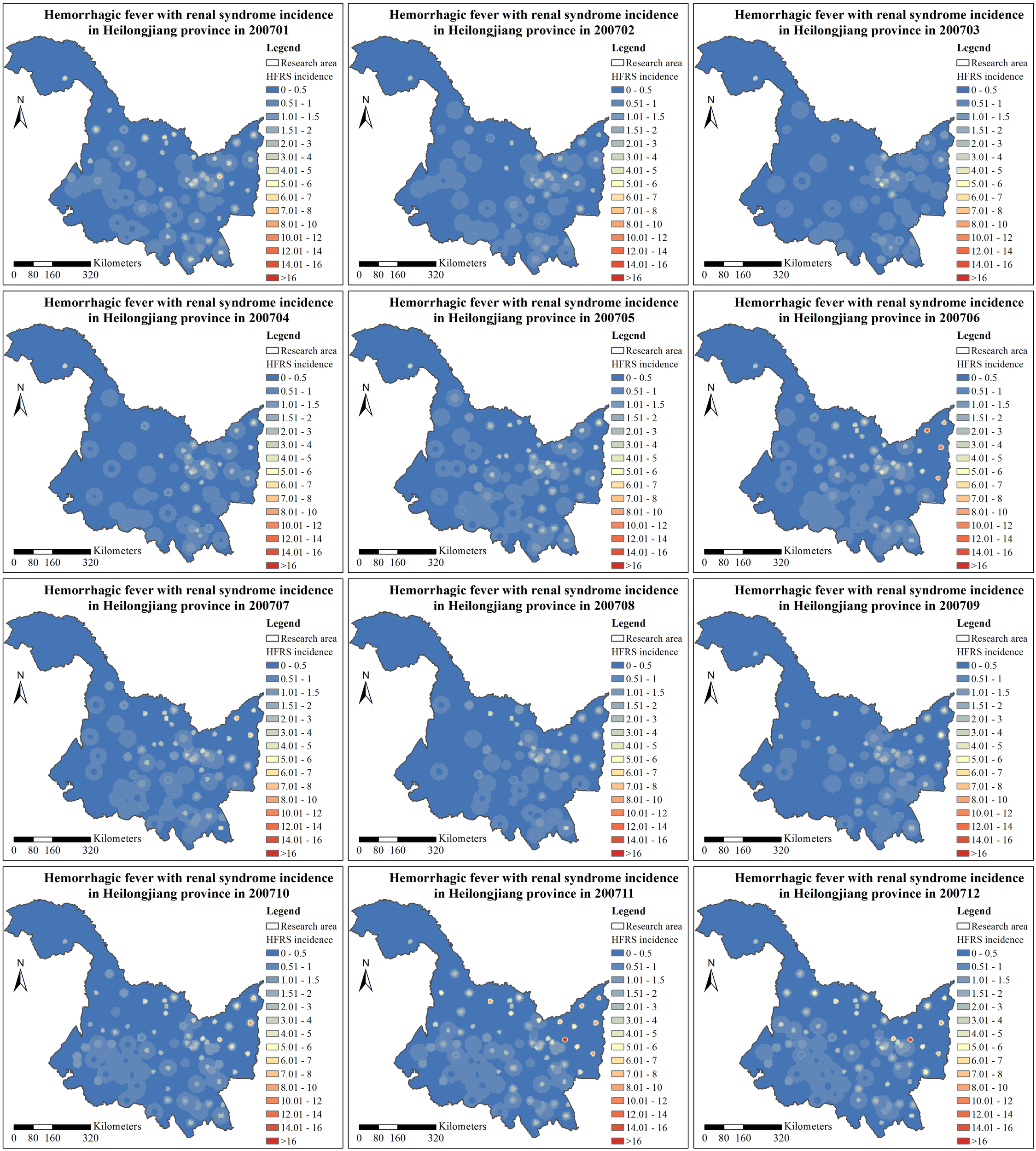

Supplement: S4 Fig — (TIF) [file pntd.0007091.s014.tif]

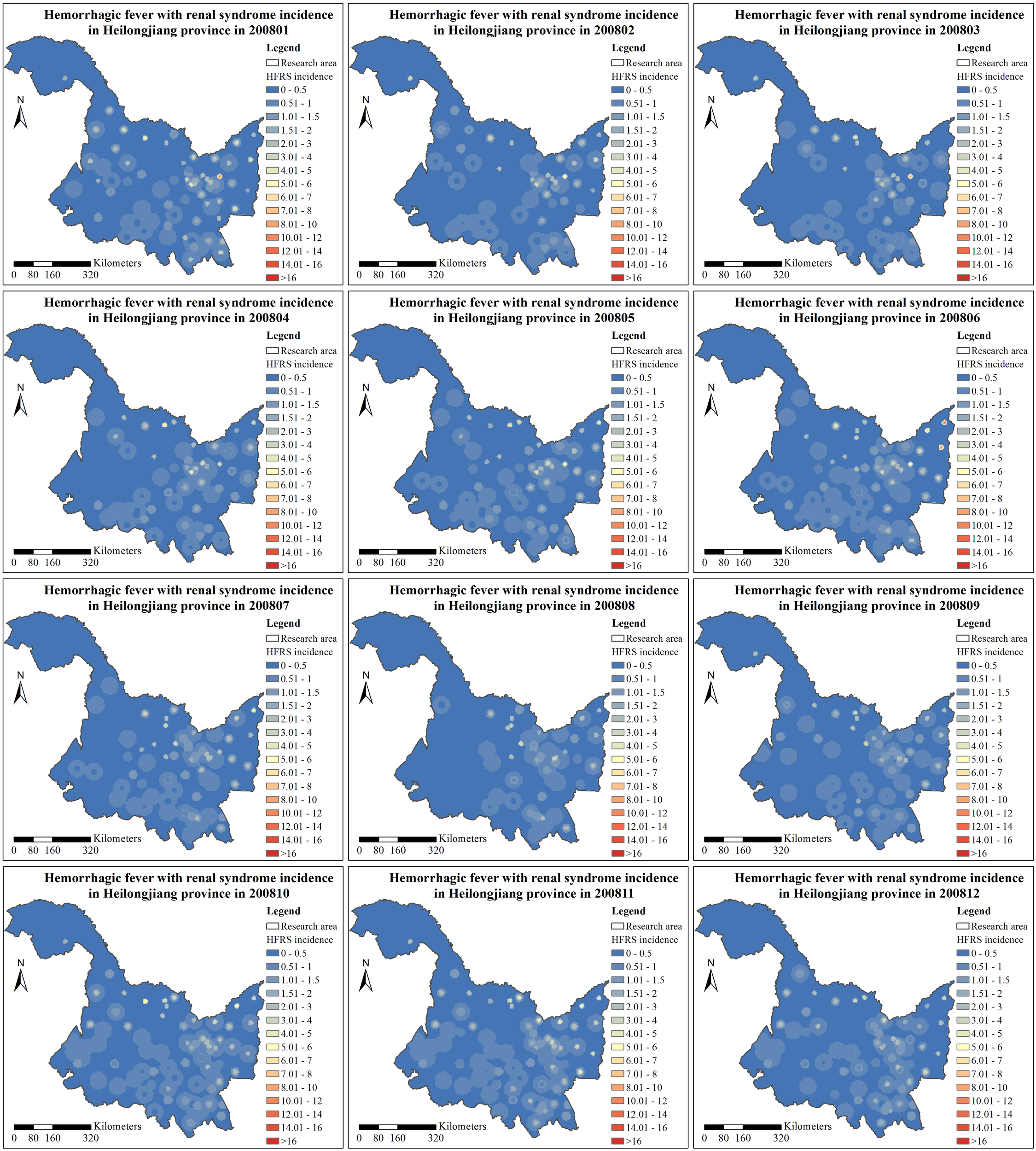

Supplement: S5 Fig — (TIF) [file pntd.0007091.s015.tif]

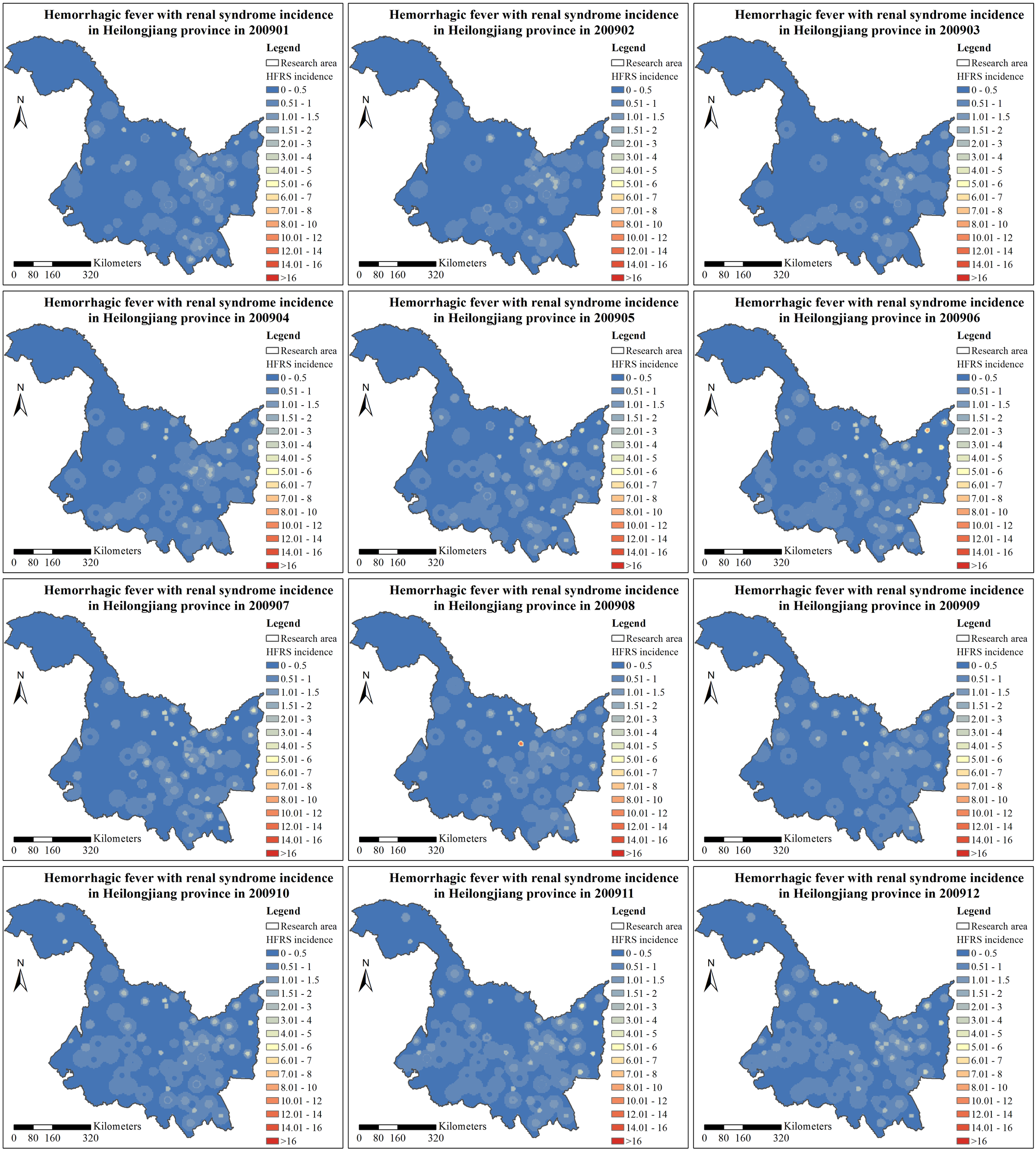

Supplement: S6 Fig — (TIF) [file pntd.0007091.s016.tif]

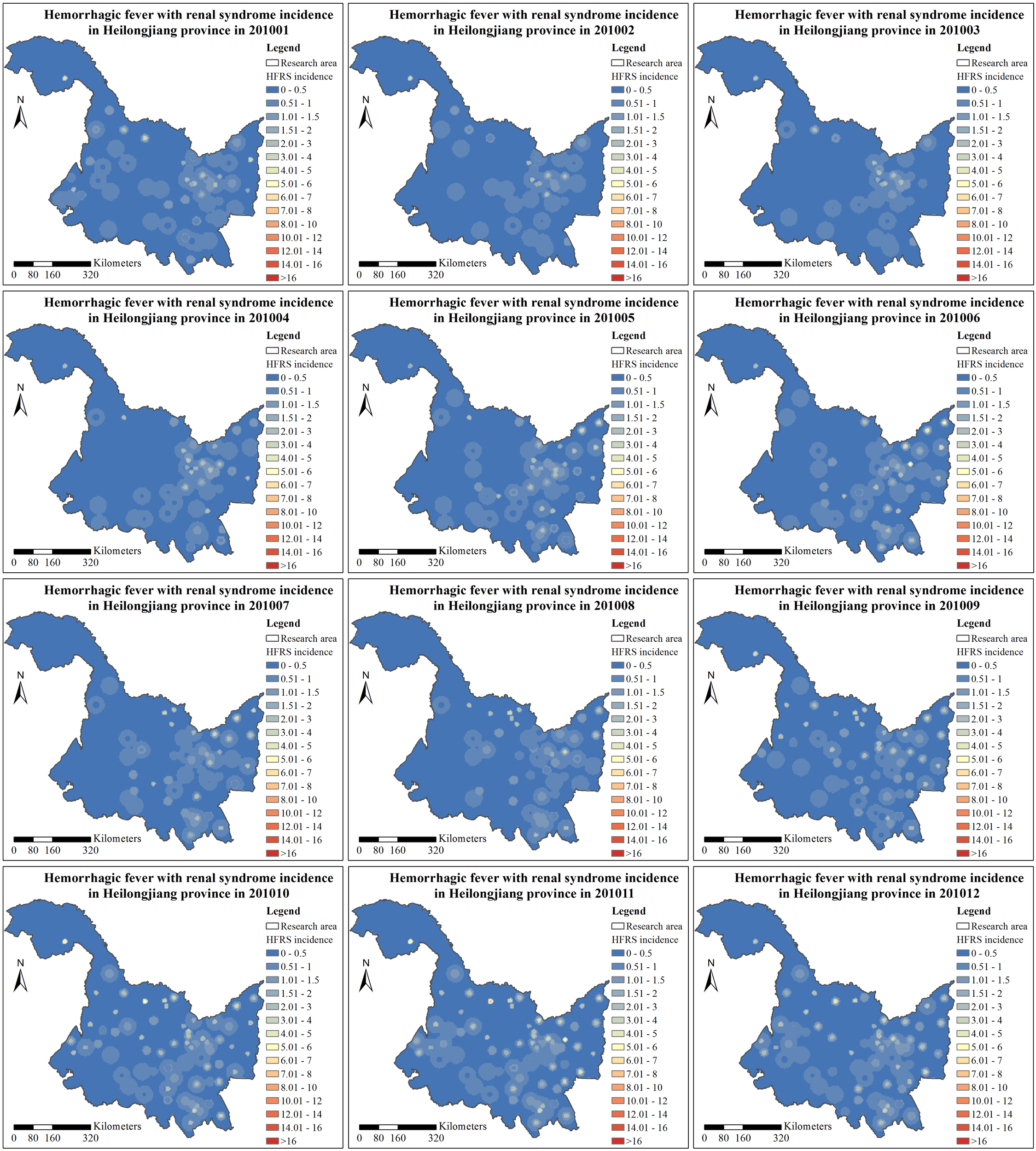

Supplement: S7 Fig — (TIF) [file pntd.0007091.s017.tif]

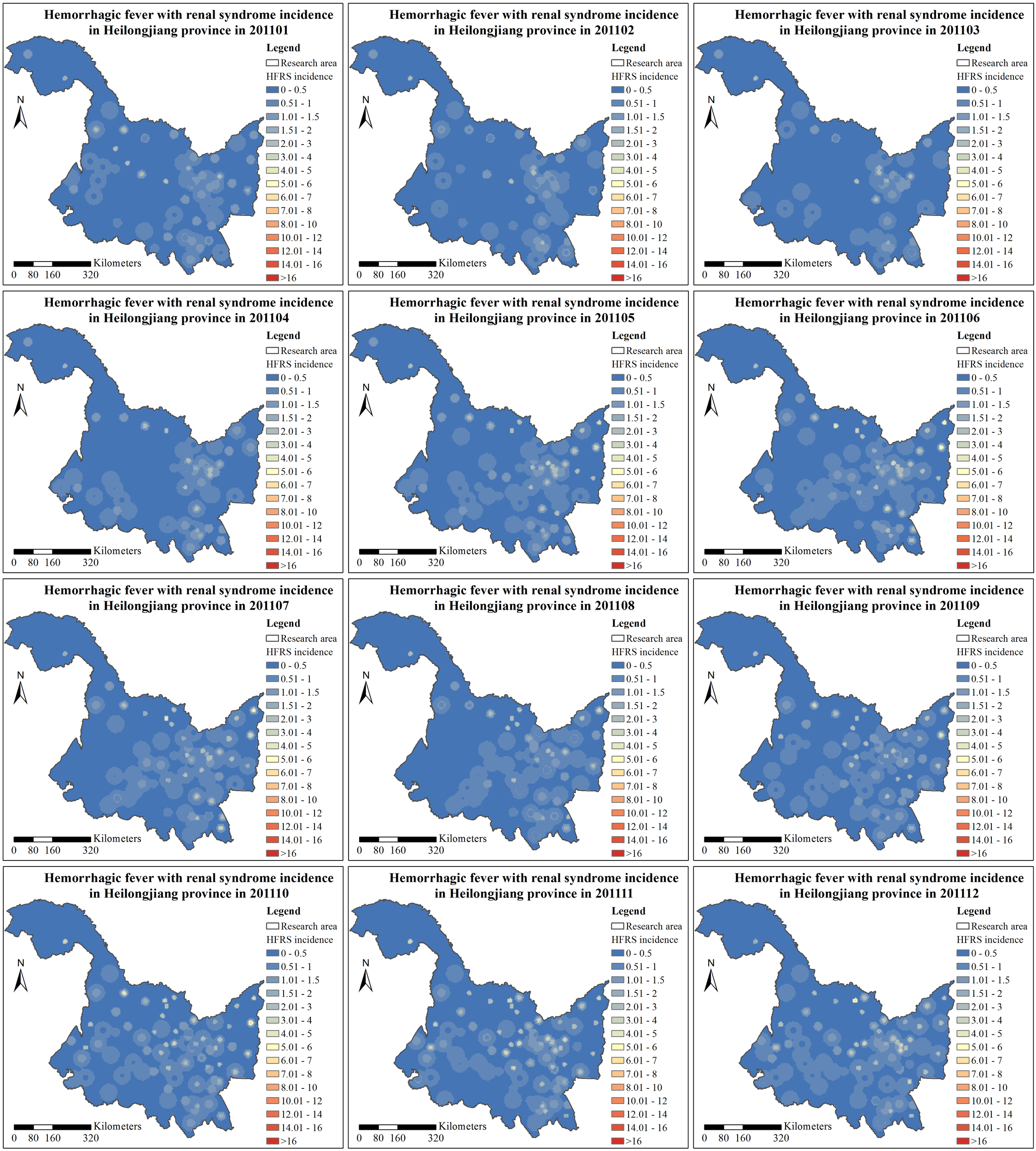

Supplement: S8 Fig — (TIF) [file pntd.0007091.s018.tif]

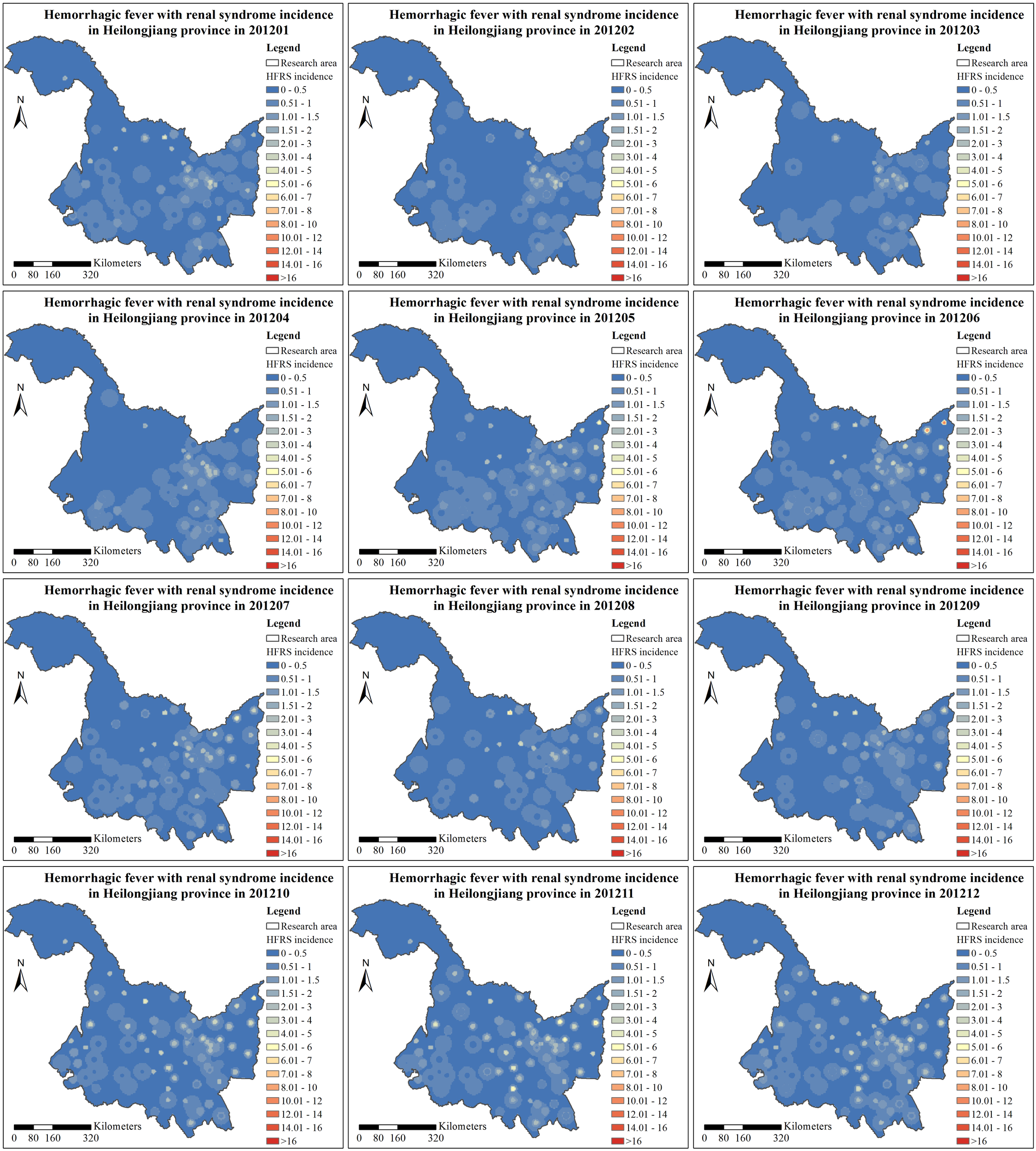

Supplement: S9 Fig — (TIF) [file pntd.0007091.s019.tif]

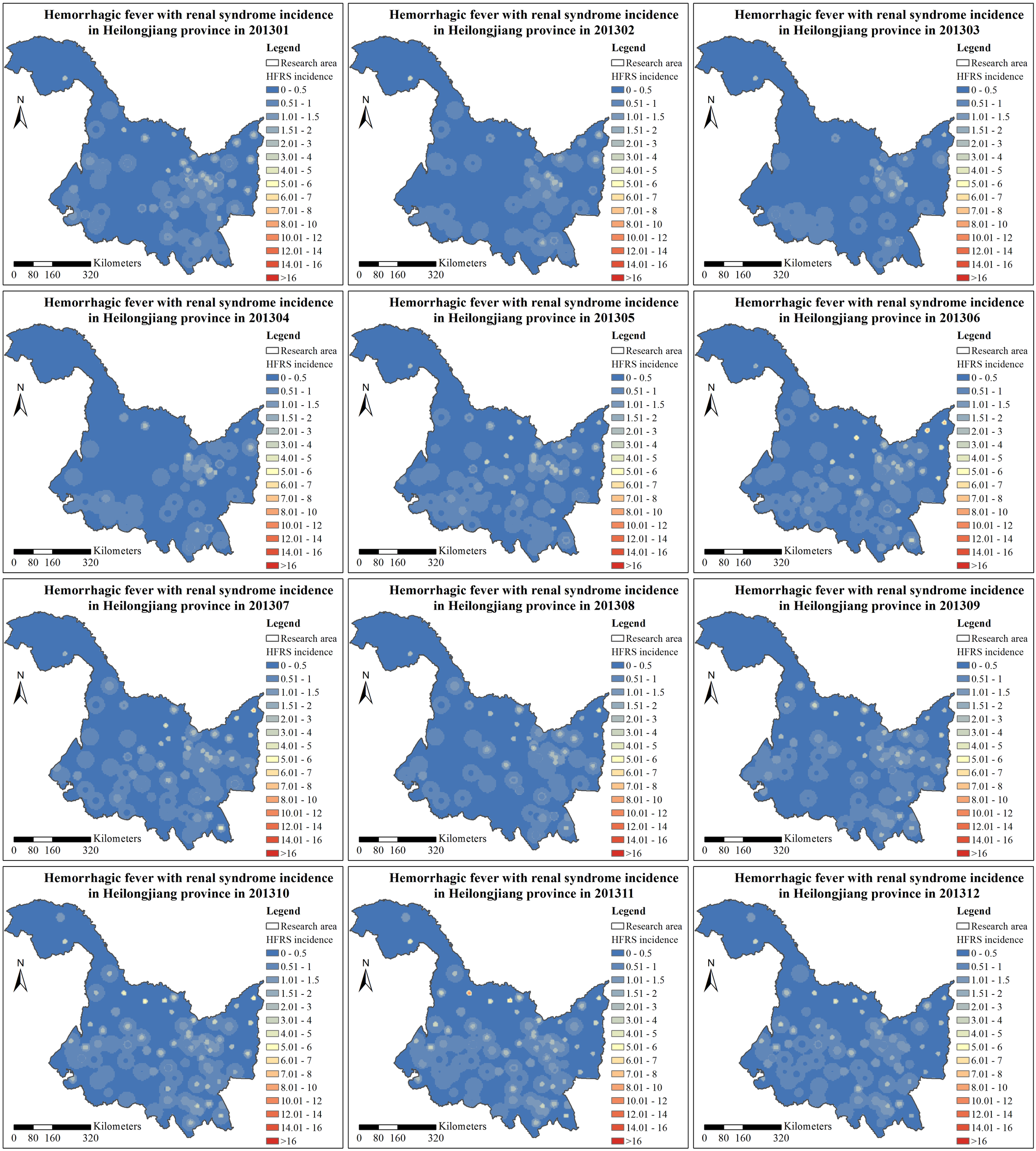

Supplement: S10 Fig — (TIF) [file pntd.0007091.s020.tif]

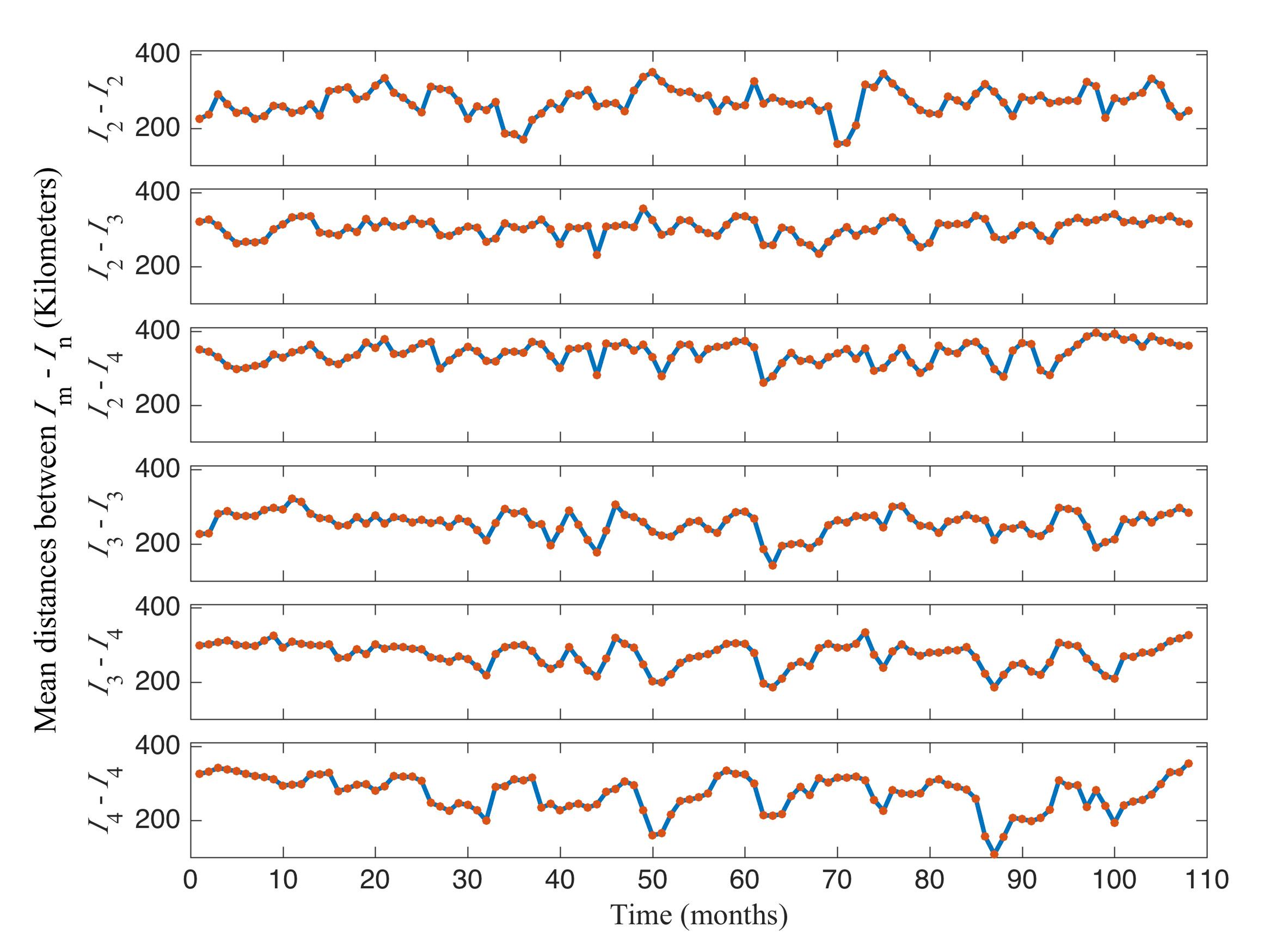

Supplement: S11 Fig — (TIF) [file pntd.0007091.s021.tif]

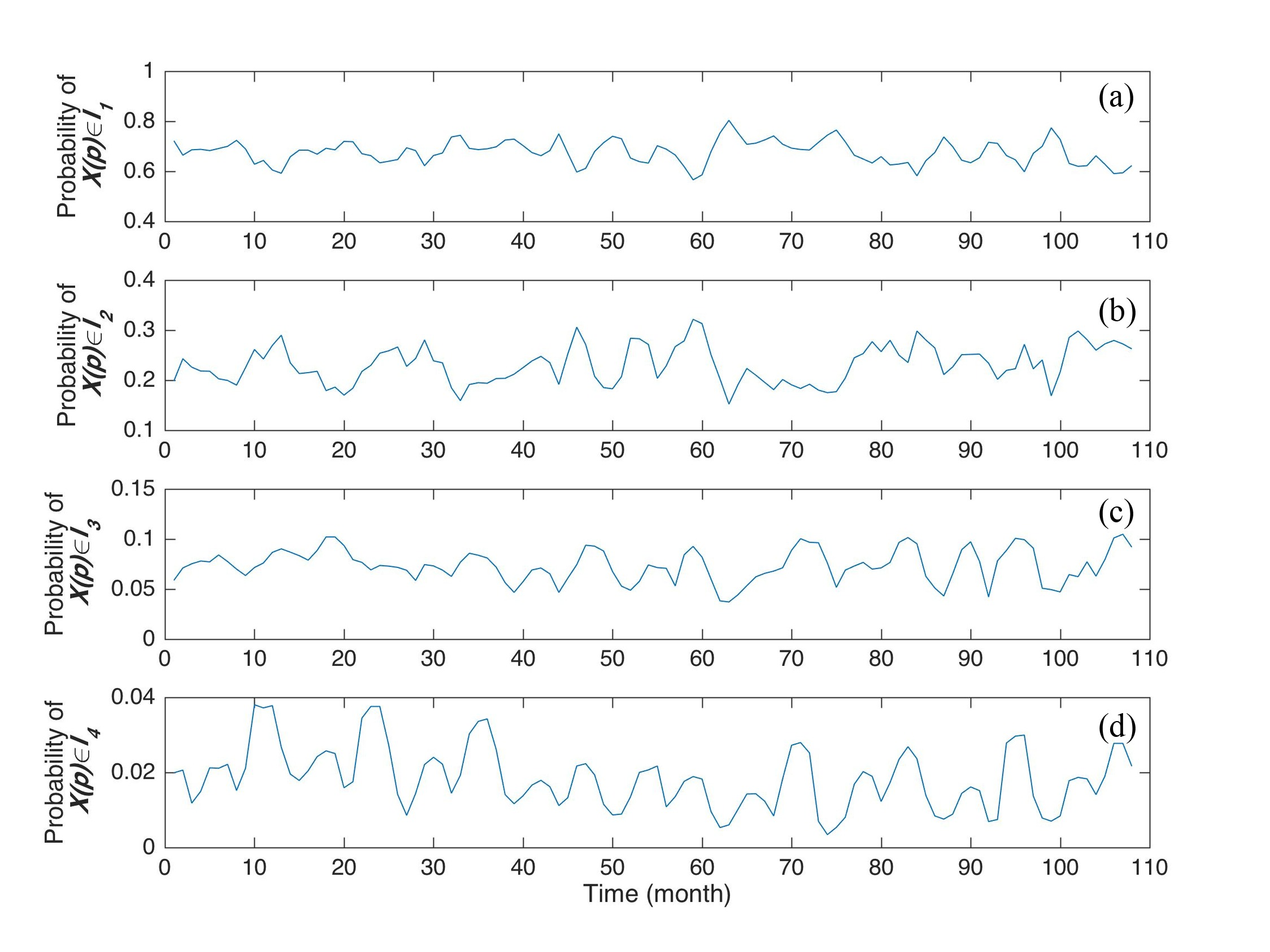

Supplement: S12 Fig — (TIF) [file pntd.0007091.s022.tif]

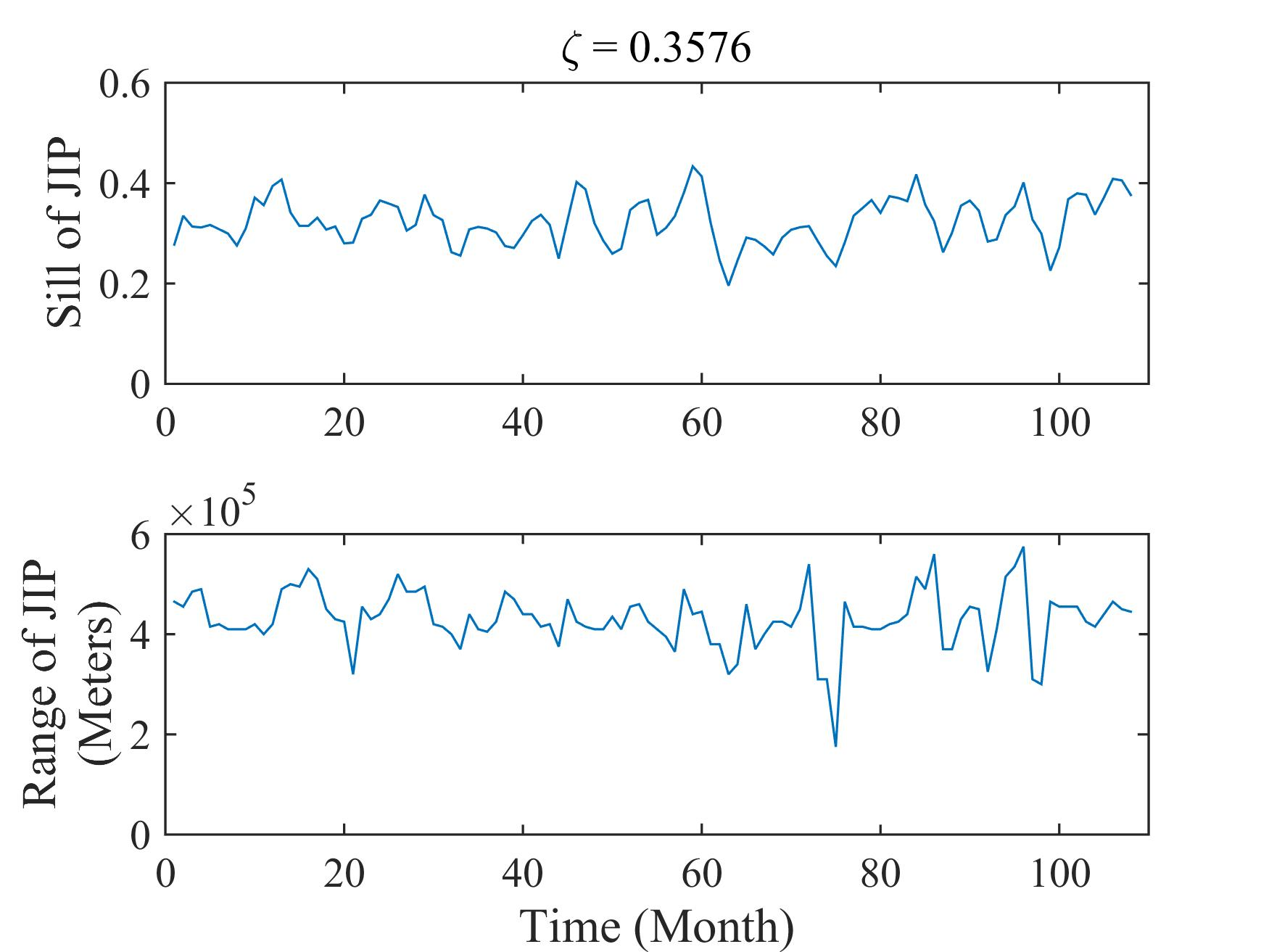

Supplement: S13 Fig — (TIF) [file pntd.0007091.s023.tif]

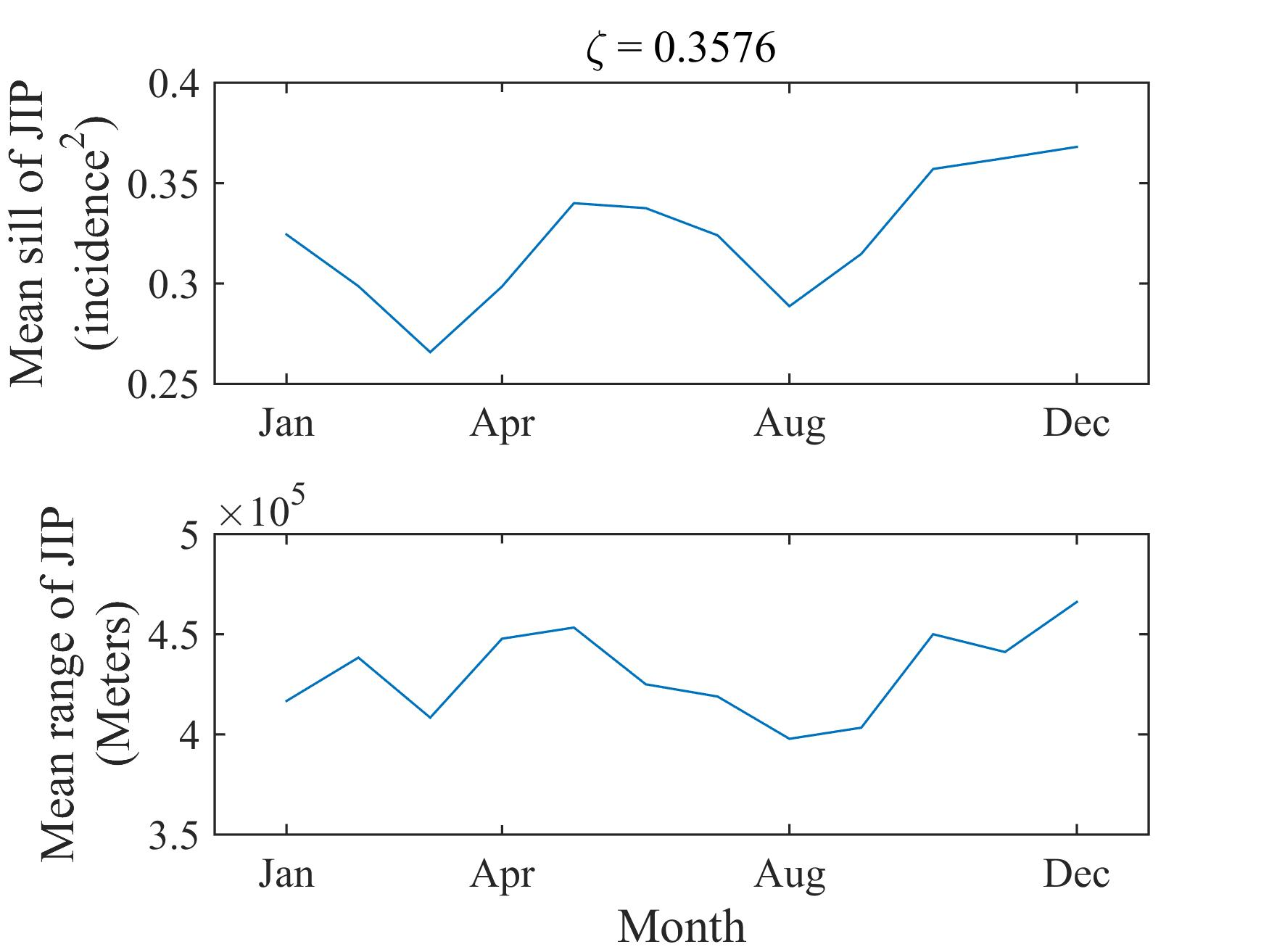

Supplement: S14 Fig — (TIF) [file pntd.0007091.s024.tif]

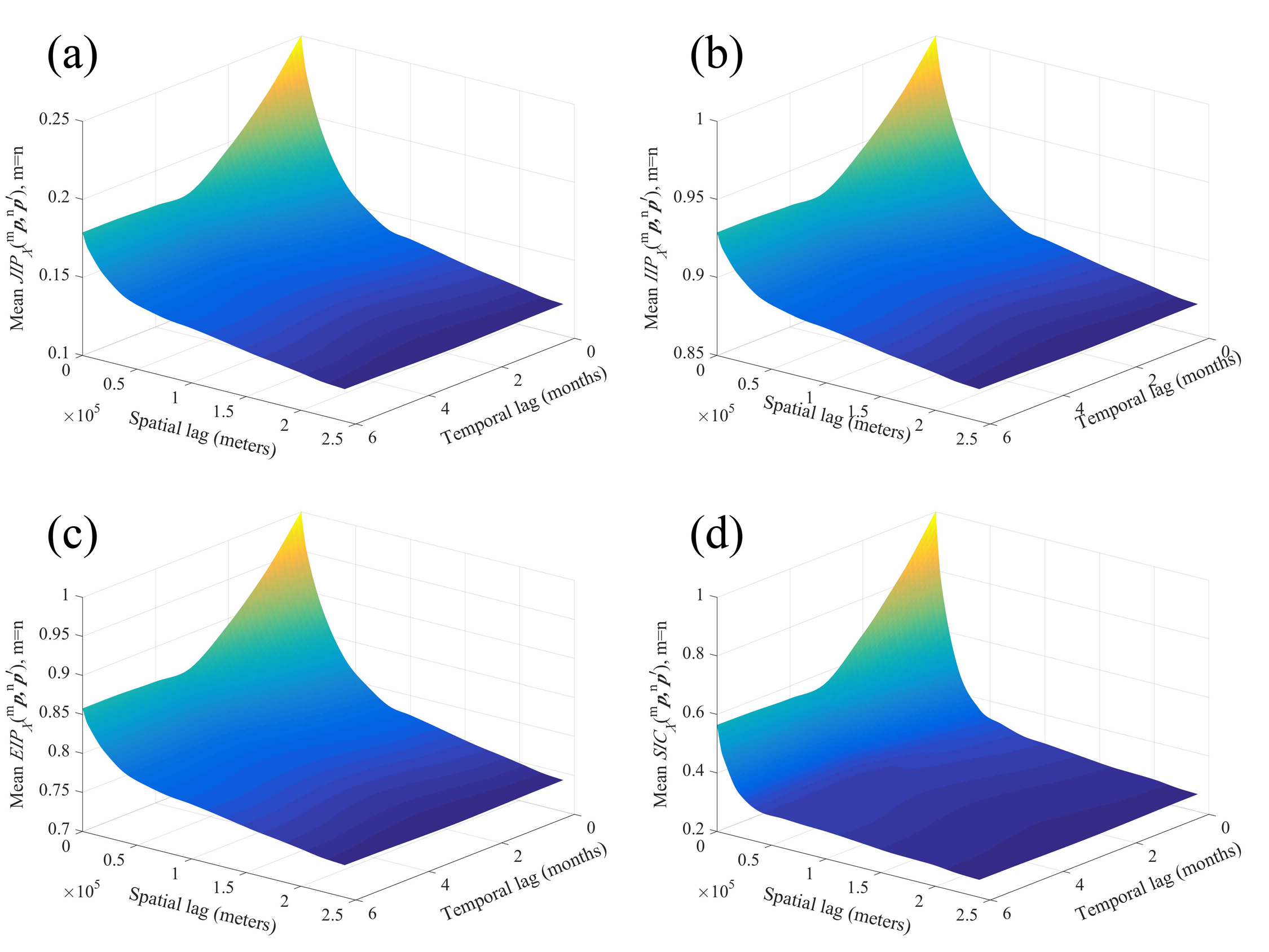

Supplement: S15 Fig — (TIF) [file pntd.0007091.s025.tif]

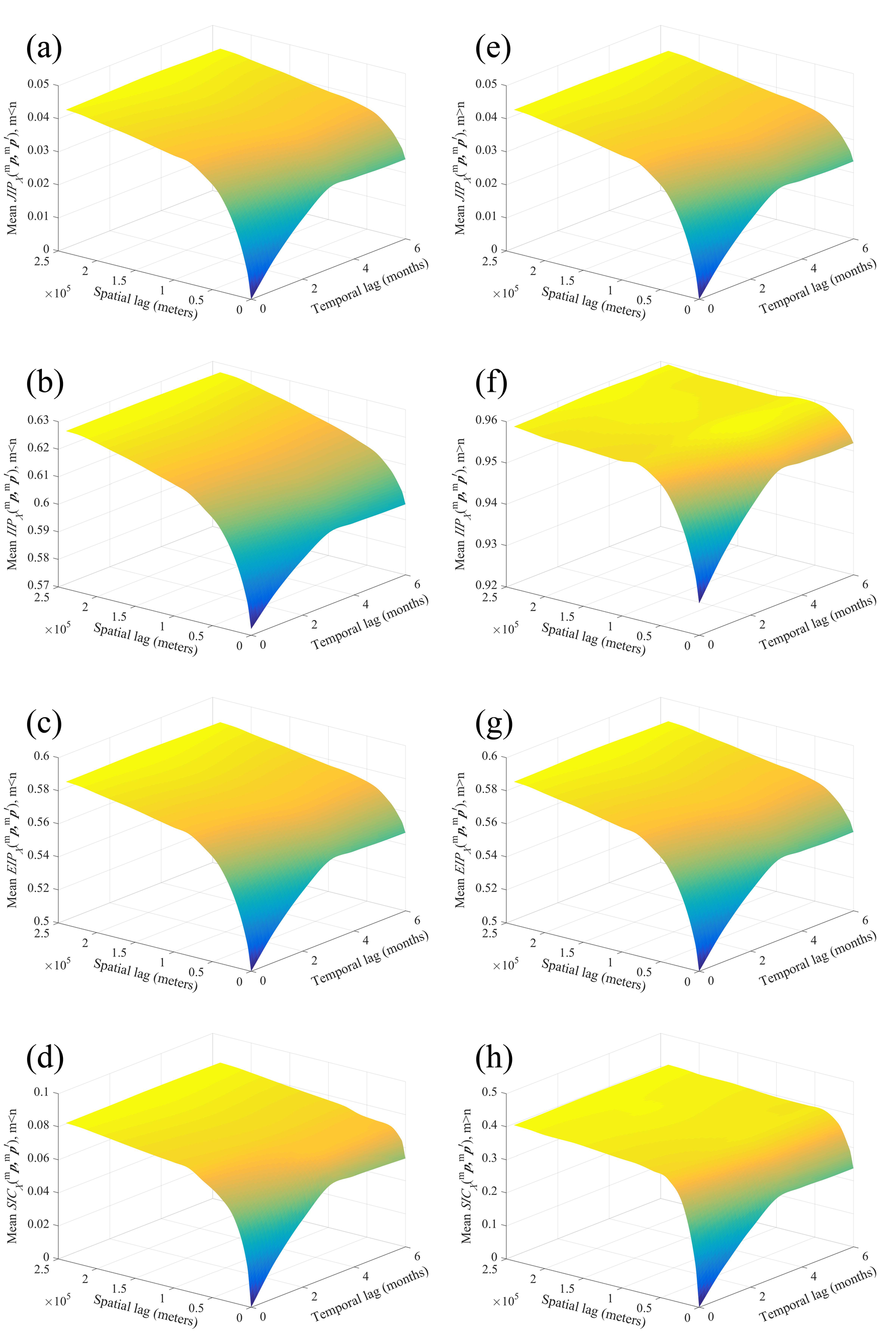

Supplement: S16 Fig — (TIF) [file pntd.0007091.s026.tif]

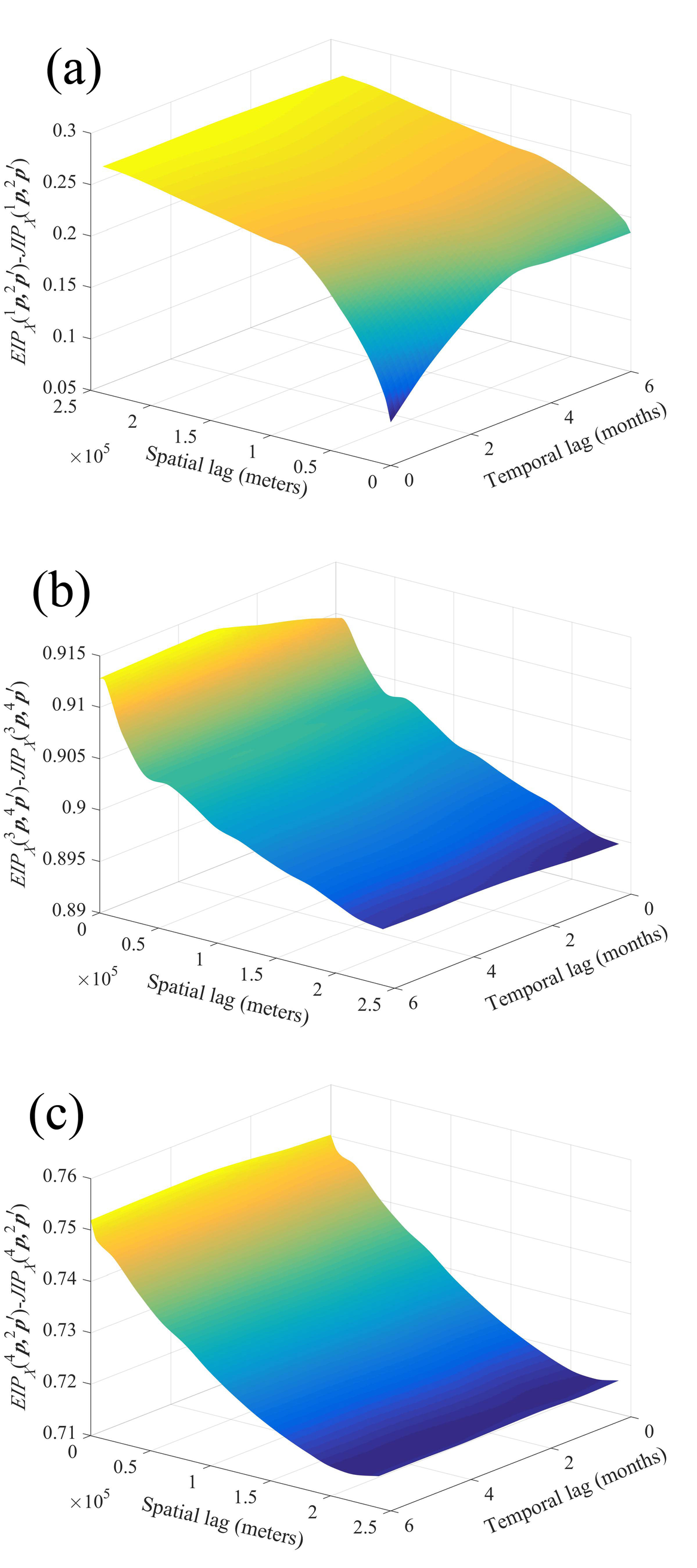

Supplement: S17 Fig — (TIF) [file pntd.0007091.s027.tif]
