# Supplementary material for: Probabilistic logic analysis of the highly heterogeneous spatiotemporal HFRS incidence distribution in Heilongjiang province (China) during 2005-2013
Source: PLoS Negl Trop Dis. 2019 Jan 31;13(1):e0007091. doi: 10.1371/journal.pntd.0007091 (PMC6380603; doi:10.1371/journal.pntd.0007091)
Supplement: S1 Table — (DOCX) [file pntd.0007091.s028.docx]

**S1 Table:** Summary statistics of HFRS incidence data in Heilongjiang province before and after log-transformation.

| *Class* | *Minimum* | *Maximum* | *Mean* | *SD* | *CV* | *Skewness* | *Kurtosis* |
| --- | --- | --- | --- | --- | --- | --- | --- |
| Original Data | 0 | 26.79 | 0.562 | 1.394 | 2.481 | 6.265 | 64.007 |
| Log-transformed Data | 0 | 1.44 | 0.125 | 0.204 | 1.630 | 2.075 | 4.714 |

*Note*: SD denotes std deviation, CV denotes coefficient of variation. The number of data points is 14040.
