# Supplementary material for: Probabilistic logic analysis of the highly heterogeneous spatiotemporal HFRS incidence distribution in Heilongjiang province (China) during 2005-2013
Source: PLoS Negl Trop Dis. 2019 Jan 31;13(1):e0007091. doi: 10.1371/journal.pntd.0007091 (PMC6380603; doi:10.1371/journal.pntd.0007091)
Supplement: S2 Table — (DOCX) [file pntd.0007091.s029.docx]

**S2 Table:** Spatial and temporal dependency ranges and fitted theoretical covariance models of the four HFRS incidence classes

| *Class No.* | *Spatial ranges* (*km*) | *Temporal ranges* (*months*) | *Fitted Covariance Model* |
| --- | --- | --- | --- |
| 1 | 10 (*Sph* model) | 2.4 (*Sph* model) | $c_{X}(\left\vert h\right\vert,\tau)=Sph(10000)Sph(2.4)$ |
| 2 | 20 (*Sph* model) | 2.6 (*Gau* model) | $c_{X}(\left\vert h\right\vert,\tau)=Sph(20000)Gau(2.6)$ |
| 3 | 30 (*Sph* model) | 2.4 (*Gau* model) | $c_{X}(\left\vert h\right\vert,\tau)=Sph(30000)Gau(2.4)$ |
| 4 | 30 (*Sph* model)  1000 (*Exp* model) | 2.3 (*Gau* model) | $\begin{matrix} c_{X}(\left\vert h\right\vert,\tau)=0.85Sph(30000)Gau(2.3) \\ \text{ }+0.15Exp(1000000)Gau(2.3) \end{matrix}$ |

Note: $|h|$ and $\tau$ are the spatial and temporal lags, respectively; *Sph*, *Gau* and *Exp* denote the standard Spherical, Gaussian, and Exponential models, respectively. These models are readily available in the relevant software libraries, like the SEKS-GUI used in this work.
