# Supplementary material for: Probabilistic logic analysis of the highly heterogeneous spatiotemporal HFRS incidence distribution in Heilongjiang province (China) during 2005-2013
Source: PLoS Negl Trop Dis. 2019 Jan 31;13(1):e0007091. doi: 10.1371/journal.pntd.0007091 (PMC6380603; doi:10.1371/journal.pntd.0007091)
Supplement: S3 Table — (DOCX) [file pntd.0007091.s030.docx]

**S3 Table:** Accuracy performance of class-dependent, standard BME and IDW implementations in HFRS incidence estimation.

| *Methods* | *RMSE* | *MAE* | *R*^2^ |
| --- | --- | --- | --- |
| *Class-dependent BME(4 classes)* | 0.74 | 0.19 | 0.72 |
| *Standard BME* | 1.11 | 0.43 | 0.44 |
| *IDW* | 1.18 | 0.53 | 0.34 |
| *Class-dependent BME (3 classes)* | 0.78 | 0.21 | 0.69 |

*Note*: The results of class-dependent BME are derived by combining the ten-fold cross validation values obtained for each class. Units of RMSE and MAE are cases/10^5^ capita. The class-dependent BME (4 classes) was employed in the present study; while the class-dependent BME (3 classes) were employed for testing the robust of class-dependent BME compared to standard BME. The IDW method was used for mapping at various time instants separately.
