# Supplementary material for: Probabilistic logic analysis of the highly heterogeneous spatiotemporal HFRS incidence distribution in Heilongjiang province (China) during 2005-2013
Source: PLoS Negl Trop Dis. 2019 Jan 31;13(1):e0007091. doi: 10.1371/journal.pntd.0007091 (PMC6380603; doi:10.1371/journal.pntd.0007091)
Supplement: S4 Table — (DOCX) [file pntd.0007091.s031.docx]

**S4 Table:** Performance of the three HFRS incidence estimation methods for each class.

| Class No. | cd-BME (4 classes) | |  | s-BME | |  | IDW | |
| --- | --- | --- | --- | --- | --- | --- | --- | --- |
|  | *RMSE* | *MAE* |  | *RMSE* | *MAE* |  | *RMSE* | *MAE* |
| 1 | 0.10 | 0.04 (65.16%) |  | 0.30 | 0.11 |  | 0.42 | 0.28 |
| 2 | 0.09 | 0.07 (82.13%) |  | 0.45 | 0.37 |  | 0.38 | 0.28 |
| 3 | 0.24 | 0.19 (72.32%) |  | 0.79 | 0.67 |  | 0.64 | 0.55 |
| 4 | 2.26 | 1.36 (42.18%) |  | 3.22 | 2.34 |  | 3.39 | 2.43 |

*Note*: The results of s-BME and IDW are obtained by dividing the 10-fold cross validation result from the entire HFRS incidence dataset into four classes according to divide process of *cd*-BME. Units of RMSE and MAE are cases/10^5^ populations; the improvement of *cd*-BME compared to *s*-BME is showed in brackets.
