# Supplementary material for: Probabilistic logic analysis of the highly heterogeneous spatiotemporal HFRS incidence distribution in Heilongjiang province (China) during 2005-2013
Source: PLoS Negl Trop Dis. 2019 Jan 31;13(1):e0007091. doi: 10.1371/journal.pntd.0007091 (PMC6380603; doi:10.1371/journal.pntd.0007091)
Supplement: S5 Table — (DOCX) [file pntd.0007091.s032.docx]

**S5 Table:** Space-time averaged JIP values of the four HFRS classes.

| $\mathbf{p}'$🡺 | $I_{1}$ | $I_{2}$ | $I_{3}$ | $I_{4}$ |
| --- | --- | --- | --- | --- |
| $\mathbf{p}$🡻 |  |  |  |  |
| $I_{1}$ | 0.4593 | 0.1344 | 0.0412 | 0.0103 |
| $I_{2}$ | 0.1323 | 0.0924 | 0.0244 | 0.0050 |
| $I_{3}$ | 0.0408 | 0.0240 | 0.0137 | 0.0026 |
| $I_{4}$ | 0.0103 | 0.0049 | 0.0025 | 0.0018 |
