# Supplementary material for: Probabilistic logic analysis of the highly heterogeneous spatiotemporal HFRS incidence distribution in Heilongjiang province (China) during 2005-2013
Source: PLoS Negl Trop Dis. 2019 Jan 31;13(1):e0007091. doi: 10.1371/journal.pntd.0007091 (PMC6380603; doi:10.1371/journal.pntd.0007091)
Supplement: S6 Table — (DOCX) [file pntd.0007091.s033.docx]

**S6 Table:** Space-time averaged IIP values of the four HFRS classes.

| $\mathbf{p}'$🡺 | $I_{1}$ | $I_{2}$ | $I_{3}$ | $I_{4}$ |
| --- | --- | --- | --- | --- |
| $\mathbf{p}$🡻 |  |  |  |  |
| $I_{1}$ | 0.8140 | 0.4892 | 0.3960 | 0.3651 |
| $I_{2}$ | 0.8783 | 0.8383 | 0.7703 | 0.7509 |
| $I_{3}$ | 0.9597 | 0.9429 | 0.9326 | 0.9214 |
| $I_{4}$ | 0.9907 | 0.9854 | 0.9830 | 0.9823 |
