# Supplementary material for: Probabilistic logic analysis of the highly heterogeneous spatiotemporal HFRS incidence distribution in Heilongjiang province (China) during 2005-2013
Source: PLoS Negl Trop Dis. 2019 Jan 31;13(1):e0007091. doi: 10.1371/journal.pntd.0007091 (PMC6380603; doi:10.1371/journal.pntd.0007091)
Supplement: S7 Table — (DOCX) [file pntd.0007091.s034.docx]

**S7 Table:** I Space-time averaged EIP values of the four HFRS classes.

| $\mathbf{p}'$🡺 | $I_{1}$ | $I_{2}$ | $I_{3}$ | $I_{4}$ |
| --- | --- | --- | --- | --- |
| $\mathbf{p}$🡻 |  |  |  |  |
| $I_{1}$ | 0.6306 | 0.3678 | 0.3553 | 0.3557 |
| $I_{2}$ | 0.3679 | 0.6749 | 0.7128 | 0.7362 |
| $I_{3}$ | 0.3578 | 0.7112 | 0.8645 | 0.9043 |
| $I_{4}$ | 0.3583 | 0.7345 | 0.9037 | 0.9644 |
