# Supplementary material for: Probabilistic logic analysis of the highly heterogeneous spatiotemporal HFRS incidence distribution in Heilongjiang province (China) during 2005-2013
Source: PLoS Negl Trop Dis. 2019 Jan 31;13(1):e0007091. doi: 10.1371/journal.pntd.0007091 (PMC6380603; doi:10.1371/journal.pntd.0007091)
Supplement: S8 Table — (DOCX) [file pntd.0007091.s035.docx]

**S8 Table:** Space-time averaged SIC values of the four HFRS classes.

| $\mathbf{p}'$🡺 | $I_{1}$ | $I_{2}$ | $I_{3}$ | $I_{4}$ |
| --- | --- | --- | --- | --- |
| $\mathbf{p}$🡻 |  |  |  |  |
| $I_{1}$ | 0.7099 | 0.2096 | 0.0644 | 0.0161 |
| $I_{2}$ | 0.5166 | 0.3682 | 0.0958 | 0.0194 |
| $I_{3}$ | 0.4974 | 0.2956 | 0.1749 | 0.0320 |
| $I_{4}$ | 0.5210 | 0.2503 | 0.1309 | 0.0979 |
