# Supplementary material for: Probabilistic logic analysis of the highly heterogeneous spatiotemporal HFRS incidence distribution in Heilongjiang province (China) during 2005-2013
Source: PLoS Negl Trop Dis. 2019 Jan 31;13(1):e0007091. doi: 10.1371/journal.pntd.0007091 (PMC6380603; doi:10.1371/journal.pntd.0007091)
Supplement: S12 Table — (DOCX) [file pntd.0007091.s039.docx]

**S12 Table:** SIC values of the four HFRS incidence classes ($\mathbf{h}=0, \tau=0$).

| $\mathbf{p}'$🡺 | $I_{1}$ | $I_{2}$ | $I_{3}$ | $I_{4}$ |
| --- | --- | --- | --- | --- |
| $\mathbf{p}$🡻 |  |  |  |  |
| $I_{1}$ | 1.0000 | 0.0000 | 0.0000 | 0.0000 |
| $I_{2}$ | 0.0000 | 1.0000 | 0.0000 | 0.0000 |
| $I_{3}$ | 0.0000 | 0.0000 | 1.0000 | 0.0000 |
| $I_{4}$ | 0.0000 | 0.0000 | 0.0000 | 1.0000 |
